# Description of *Streptomyces naphthomycinicus* sp. nov., an endophytic actinobacterium producing naphthomycin A and its genome insight for discovering bioactive compounds

**DOI:** 10.3389/fmicb.2024.1353511

**Published:** 2024-04-17

**Authors:** Onuma Kaewkla, Mike Perkins, Arinthip Thamchaipenet, Weerachai Saijuntha, Sudarat Sukpanoa, Chanwit Suriyachadkun, Nitcha Chamroensaksri, Theeraphan Chumroenphat, Christopher Milton Mathew Franco

**Affiliations:** ^1^Center of Excellence in Biodiversity Research, Mahasarakham University, Maha Sarakham, Thailand; ^2^Department of Medical Biotechnology, College of Medicine and Public Health, Flinders University, Adelaide, SA, Australia; ^3^Deparment of Chemistry, College of Science and Engineering, Flinders University, Adelaide, SA, Australia; ^4^Department of Genetics, Kasetsart University, Bangkok, Thailand; ^5^Faculty of Medicine, Mahasarakham University, Maha Sarakham, Thailand; ^6^Department of Biology, Faculty of Science, Mahasarakham University, Maha Sarakham, Thailand; ^7^Thailand Bioresource Research Center (TBRC), National Center for Genetic Engineering and Biotechnology, National Science and Technology Development Agency, Pathumthani, Thailand; ^8^National Biobank of Thailand (NBT), National Center for Genetic Engineering and Biotechnology, National Science and Technology Development Agency, Pathumthani, Thailand; ^9^Aesthetic Sciences and Health Program, Faculty of Thai Traditional and Alternative Medicine, Ubon Ratchathani Rajabhat University, Ubon Ratchathani, Thailand

**Keywords:** *Streptomyces naphthomycinicus*, endophyte, naphthomycin A, solid-state fermentation, genome insight

## Abstract

Endophytic actinobacteria are a group of bacteria living inside plant tissue without harmful effects, and benefit the host plant. Many can inhibit plant pathogens and promote plant growth. This study aimed to identify a strain of *Streptomyces* as a novel species and study its antibiotics production. An endophytic actinobacterium, strain TML10^T^ was isolated from a surface-sterilized leaf of a Thai medicinal plant (*Terminalia mucronata* Craib and Hutch). As a result of a polyphasic taxonomy study, strain TML10^T^ was identified as a member of the genus *Streptomyces*. Strain TML10^T^ was an aerobic actinobacterium with well-developed substrate mycelia with loop spore chains and spiny surface. Chemotaxonomic data, including cell wall components, major menaquinones, and major fatty acids, confirmed the affiliation of strain TML10^T^ to the genus *Streptomyces*. The results of the phylogenetic analysis, including physiological and biochemical studies in combination with a genome comparison study, allowed the genotypic and phenotypic differentiation of strain TML10^T^ and the closest related type strains. The digital DNA-DNA hybridization (dDDH), Average nucleotide identity Blast (ANIb), and ANIMummer (ANIm) values between strain TML10^T^ and the closest type strain, *Streptomyces musisoli* CH5-8^T^ were 38.8%, 88.5%, and 90.8%, respectively. The name proposed for the new species is *Streptomyces naphthomycinicus* sp. nov. (TML10^T^ = TBRC 15050^T^ = NRRL B-65638^T^). Strain TML10^T^ was further studied for liquid and solid-state fermentation of antibiotic production. Solid-state fermentation with cooked rice provided the best conditions for antibiotic production against methicillin-resistant *Staphylococcus aureus*. The elucidation of the chemical structures from this strain revealed a known antimicrobial agent, naphthomycin A. Mining the genome data of strain TML10^T^ suggested its potential as a producer of antbiotics and other valuable compounds such as ε-Poly-L-lysine (ε-PL) and arginine deiminase. Strain TML10^T^ contains the *arc*A gene encoding arginine deiminase and could degrade arginine *in vitro*.

## Introduction

Endophytes are an endosymbiotic group of microorganisms that inhabit plant tissues. They can be isolated from surface sterilized plants using microbial or plant growth medium and benefit as reservoirs of novel bioactive secondary metabolites (Golinska et al., 2015). Endophytic bacteria live inside plant tissues such as stems, roots, leaves, tubers, and fruits without any harmful effects, and they are found in a variety of woody, horticultural, shrub, and ornamental plants (Tripathi et al., 2022). Currently, there are many reports of endophytic bacteria and their applications in the agricultural and biotechnological arena (Eid et al., 2021; Wu et al., 2021; Li et al., 2023). Endophytic bacteria benefit plants as plant growth-promoting bacteria (PGPB) such as fixing nitrogen, producing siderophores, solubilizing inorganic phosphate, and producing phytohormones such as auxin, cytokinin and gibberellin (Mushtaq et al., 2023). They can help to protect their host plants by producing antibiotic compounds and lytic enzymes to inhibit plant pathogens and also can induce systematic resistance (ISR) or systematic acquire resistance (SAR) (Mishra et al., 2022; Li et al., 2023). Selected source plants are essential for a program targeting the isolation of rare and novel endophytic actinobacteria, which are likely to produce novel compounds (Kaewkla and Franco, 2013; Golinska et al., 2015). The problems of the increasing occurrence of multi-resistance pathogens, the evolution of new diseases, and the toxicity of currently used compounds lead to the discovery, production, and marketing of new antibiotics (Rokem et al., 2007). In the past 10 years, researchers have been interested in isolating *Streptomyces* from plant tissue, as they are a good source of plant growth-promoting activity and a variety of bioactive compounds (Gao et al., 2018; Wu et al., 2021). The genus *Streptomyces* belongs to the family *Streptomycetaceae*, containing more than 650 validly published species (https://lpsn.dsmz.de/genus/Streptomyces; accessed on 20 July 2023) (Parte et al., 2020). Most of them were isolated from soil but can also be isolated from diverse environments, including plant tissue.

Endophytic *Streptomyces* strains are known to control plant pathogens and weeds. For example, *Streptomyces* sp. MSU-2110 produced coronamycins, peptide antibiotics against pythiaceous fungi (Ezra et al., 2004), and *Streptomyces* sp. SANK 63997 produced herbicidin A, B, and H (Kizuka et al., 1998). They are also well-known for synthesizing novel bioactive compounds such as anticancer agents pterocin (Igarashi et al., 2006), daidzein, and *N*-acetyltyramine (Liu et al., 2009). Some strains produced known but essential bioactive compounds such as the antitumor agent, neoechinulin, from *Streptomyces* sp. LRE541 (Ma et al., 2021). Although *Streptomyces* spp. are well known as producers of a variety of secondary metabolites, many compounds predicted through analysis of biosynthetic gene clusters (BGCs) in the genome are not produced under normal laboratory conditions. Therefore, these cryptic BGCs require to be activated and manipulated by optimization of culture and fermentation conditions, heterologous expression, co-culture cultivation, induction by signaling molecules, or activation of transcription activators (Li et al., 2021). There are several regulatory mechanisms involved in the biosynthesis of antibiotic production, including inducers (Rius et al., 1996), carbon catabolite regulation (CCR) (Rafieenia, 2013), regulation by nitrogen sources (Shapiro, 1989), inorganic phosphorus regulation (Sanchez and Demain, 2002), feedback inhibition (Sanchez and Demain, 2002), and other factors such as optimum temperature. Incubation time are also crucial for antibiotic production.

In *Streptomyces griseus*, the first signaling molecule to be discovered and studied was A-factor, C_13_ γ-butyrolactone compound. It was found to regulate the production of streptomycin and spores through a regulatory cascade involving, the receptor *arp*A (A-factor receptor protein), the pleiotropic regulator *adp*A (A-factordependent protein), and the CSR activator *str*R (specific transcriptionalactivator gene) (Horinouchi and Beppu, 2007). In *Streptomyces avermitilis*, the biosynthesis of the anthelmintic agent, avermectin requires a signaling molecule called avenolide as an inducer (Kitani et al., 2011). The regulation of carbon in cephamycin C biosynthesis by *Streptomyces clavuligerus* was reported by Lebrihi et al. (1988). Glycerol was found to repress the enzymes cephamycin C synthetase and expandase, and expandase activity was inhibited by phosphorylated intermediates of glycolysis such as glucose 6-phosphate and fructose 1–6 bis-phosphate. Nitrogen source was also important for antibiotic production. High ammonium concentrations inhibited the formation of actinorhodin in *S. coelico*lor (Hobbs et al., 1992) and the production of pristinamycin in *S. pristinaespiralis* (Voelker and Altaba, 2001).

The co-culture method is a prevalent approach to elicit cryptic biosynthetic pathways of *Streptomyces*. For example, co-culturing *Streptomyce coelicolor* M145 with other actinomycete strains showed the production of at least 12 different types of desferrioxamine (Traxler et al., 2013). Co-cultivating *Streptomyces leeuwenhoekii* C34 with *Aspergillus fumigatus* MR2012 produced a new luteoride derivative and a new pseurotin derivative, while co-cultivating *S. leeuwenhoekii* C58 with *A. fumigatus* MR2012 led to the production of a lasso peptide chaxapeptin, which displayed a significant inhibitory effect on a human lung cancer cell line (Elsayed et al., 2015; Wakefield et al., 2017).

Furthermore, liquid-state fermentation can negatively affect antibiotic production in a non-sporulating state, which may accelerate programmed cell death (PCD) (Yagüe et al., 2013). It was stated that *Streptomyces* growing in liquid cultures would develop a first mycelium stage (MI), PCD, and the differentiation of a secondary metabolite, producing mycelium (MII). For example, *Streptomyces coelicolor* A3(2) produced actinorhodin/undecylprodigiosin in liquid cultures at the stage of developing MII and this strain did not produce spore on liquid culture (Manteca et al., 2008).

In our previous studies, we explored the diversity of endophytic actinobacteria from medical plants and identified several endophytic actinobacteria as new species. The objective of this study is to describe strain TML10^T^, which was isolated from a Thai medicinal plant's surface-sterilized leaf, as a new species using a polyphasic taxonomic approach that includes genotypic and phenotypic data. This study also aims to investigate the production of antibiotics from strain TML10^T^ in liquid and solid media, determine the structures of its bioactive compounds, and examine the biosynthesis gene clusters and genome data mining of this strain. Additionally, we conducted a preliminary screening of arginine degradation of strain TML10^T^ and reported a phylogenetic tree and sequence analysis of arginine deiminase protein from strain TML10^T^ and other closely related *Streptomyces* strains. The findings of this study demonstrate the importance of selecting the appropriate media for antibiotic screening programs and the effectiveness of genome data mining in identifying potential bioactive compound producers.

## Materials and methods

### Isolation of strain TML10^*T*^

*Streptomyces* sp. strain TML10^T^ was isolated from the surface-sterilized leaf of a Thai medicinal plant (*Terminalia mucronata* Craib and Hutch). A Thai medicinal plant was collected from the Phujongnayoi National Park, Nachalui district, Ubonratchathani province, Thailand (14.438954 N 105.344589 E) and processed within 4 h of collection (Kaewkla and Franco, 2013). Briefly, the plant sample was surface sterilized using 70% ethanol for 5 min, soaked in 6% sodium hypochlorite solution (6% available chlorine and freshly prepared) for 5 min, then washed in sterile RO water five times to remove the chemicals. The sample was soaked in 10% (w/v) NaHCO_3_ for 10 min to disrupt the plant tissue and retard the growth of endophytic fungi, according to the method described by Kaewkla and Franco (2013). Crushed surface-sterilized leaf tissues were placed onto three isolation media; Humic acid vitamin B agar (HVA) (Hayakawa and Nonomura, 1987), VL70 gellan gum with the amino acid mixture and VL70 gellan gum with carboxymethyl cellulose (Schoenborn et al., 2004; Kaewkla and Franco, 2013). The media were supplemented with 20 μg ml^−1^ nalidixic acid and 100 U ml^−1^ nystatin to inhibit the growth of some bacteria and fungi, respectively. Plates were kept in plastic-sealed boxes containing wet paper towels to maintain moisture and incubated at 37 and 27°C for 12 weeks. Actinobacterium-like strains were purified using half-strength potato dextrose agar (HPDA) and preserved on HPDA slants at 4°C for short-term storage and 20% glycerol at −80°C for long-term storage (Schoenborn et al., 2004).

### 16s rRNA gene analysis and phylogenetic characteristics

Extraction of genomic DNA, 16S rRNA gene amplification, and sequencing of strain TML10^T^ were carried out as described previously (Kaewkla and Franco, 2013). The 16S rRNA gene sequence of strain TML10^T^ was analyzed using EzTaxon-e server (https://www.ezbiocloud.net/) (Yoon et al., 2017) and subsequently aligned with the representatives of the most closely related *Streptomyces* type strains using CLUSTAL X (Thompson et al., 1997) with *Embleya hyalinus* NBRC 13850^T^ as the outgroup. All sequences were trimmed to the same length. The phylogenetic trees were constructed by the maximum likelihood (ML) algorithm (Saitou and Nei, 1987) using the software package MEGA version 11 (Tamura et al., 2021). The evolutionary distances were computed using the Kimura 2-parameter method (Kimura, 1980). The topology of the tree was evaluated by performing a bootstrap analysis based on 1,000 replications (Felsenstein, 1985).

### Whole genome sequencing, and genome assembly and annotation of strain TML10^*T*^

The genomic DNA of strain TML10^T^ was extracted for whole genome sequencing by a short read platform using a previously described protocol (Saito and Miura, 1963).

A PCR-free library was prepared and sequenced as described by the previous protocol (Kaewkla et al., 2022). The reads were *de novo* assembled using Unicycler (0.4.8) (Wick et al., 2017). For the long-read technique, sequencing libraries were prepared by using a Rapid sequencing gDNA-barcoding (SQK-RBK004) kit and sequenced with long-read Oxford Nanopore MinION Mk1C (Oxford Nanopore Technologies (ONT), Oxford, UK) (Coster et al., 2018) at the Omics Sciences and Bioinformatics Center, Chulalongkorn University, Thailand. The hybrid *de novo* genome assembly pipeline—Nanopore draft Illumina polishing was carried out. First, porechop (Version 0.2.4) was used to clean adapters, and filtlong (version 0.2.1) (Wick, 2017a,b) was used to filter subreads. Subsequently, filtered reads were inputted for SPAdes (Version 3.15.4) (Antipov et al., 2015); a genome assembly program with default parameters and *de novo* genome assembly from Illumina short reads was applied as trusted contigs. Quast (Version 5.2.0) was used for genome assembly quality (Gurevich et al., 2013). BUSCO (Version 5.4.6) was used to quantitatively assess the completeness of a genome assembly (Simão et al., 2015). Prokka version 1.14.6 (Seemann, 2014) was applied to annotate the genome sequences. The draft genome assemblies of strain TML10^T^ was submitted to GenBank under Accession No. JAINRE000000000.

### Genome comparison study

Genome of strain TML10^T^ was evaluated for the average nucleotide identity (ANI) values with their related species with pairwise genome alignment by using ANI-Blast (ANIb) and ANI-MUMmer (ANIm) algorithms within the JspeciesWS web service (Richter and Rosselló-Móra, 2009; Richter et al., 2016; Nouioui et al., 2018). The phylogenetic tree of the genomes of strain TML10^T^ and their related taxa was constructed by using the Type (strain) Genome Server (TYGS) (Meier-Kolthoff et al., 2013; Meier-Kolthoff and Göker, 2019). The tree was inferred with FastME 2.1.6.1 (Lefort et al., 2015) from Genome BLAST Distance Phylogeny (GBDP) distances calculated from genome sequences. The GBDP distance formula *d4* was applied to scale branch lengths of the genomes of these two strains. The Genome-to-Genome Distance calculator (GGDC 2.1; BLAST + method) applying formula 2 (identities/HSP length) was applied to calculate digital DNA-DNA hybridization (dDDH) values of these two strains with their closely related type strains (Meier-Kolthoff et al., 2013). The maximum likelihood (ML) phylogenomic tree of the genomes of strain TML10^T^ and closely related strains with valid names of *Streptomyces*, with *Embleya hyaline* NBRC 13850^T^ as the outgroup was constructed using the codon tree option in the PATRIC web server (Wattam et al., 2017), which was based on aligned amino acids and nucleotides derived from 500 single copy genes in the genome dataset matched against the PATRIC PGFams database (http://www.patricbrc.org/) using the RAxML algorithm (Stamatakis, 2014).

### Chemotaxonomic characterization

Whole-cell sugar of strain TML10^T^ was analyzed by the TLC method of Hasegawa et al. (1983), and diaminopimelic acid (DAP) was detected by TLC using the method of O'Donnell et al. (1985). The phospholipids were analyzed as described by Minnikin et al. (1984) and Komagata and Suzuki (1987) using six spray reagents according to the previous study (Kaewkla et al., 2022). Extraction and purification of isoprenoid quinones have followed the method of Minnikin et al. (1984) with analysis of the samples as described by Kaewkla et al. (2022). For the analysis of whole-cell fatty acids, strain TML10^T^ was grown for seven days at 27°C in Tryptic Soy Broth (Oxoid) in an Erlenmeyer flask at 125 rpm and harvested by centrifugation. The fatty acid methyl esters (FAMEs) were extracted from freeze-dried cells (~50 mg) and analyzed by following the protocols described by Microbial Identification Inc. (MIDI) (Sasser, 2001). The Sherlock RTSBA6 software version 6.4 was used for analysis.

### Phenotypic characterization

Phenotypic characteristics of strain TML10^T^ and four related type strains: *Streptomyces musisoli* CH5-8^T^, *Streptomyces echinatus* JCM 4144^T^, *Streptomyces corchorusii* NBRC 13032^T^, and *Streptomyces cellostaticus* NBRC 12849^T^ were compared.

Strain TML10^T^ and its related type strain, *S. cellostaticus* NBRC 12849^T^ were grown on eight different media: yeast extract malt extract agar (ISP 2), oatmeal agar (ISP 3), inorganic salt starch agar (ISP 4), glycerol asparagine agar (ISP 5), and tyrosine agar (ISP 7) (ISP; The International *Streptomyces* Project; Shirling and Gottlieb, 1966), Bennett's agar, HPDA, and nutrient agar (Atlas and Parks, 1993). Acid production from 18 carbohydrates, decomposition of skim milk, esculin, hippurate, *L*-tyrosine, xanthine, and urea, hydrolysis of starch, assimilation of six organic acids, utilization of four phenolic compounds as sole carbon sources, and catalase production were described by Kaewkla et al. (2022). Growth at different temperatures at 4, 15, 27, 37, 45, and 55°C, growth at different pH between 4 and 10 (in 1 pH interval) and NaCl concentrations (1, 3, 5, 10, 15, and 20%, w/v) were evaluated after incubation at 27°C for 10–14 days on ISP 2 medium (Kaewkla et al., 2022).

The vegetative and aerial hyphae of strain TML10^T^ were visualized after seven days of incubation at 27°C on HPDA by scanning electron microscopy (Carl Zeiss, AURIGA), and the preparation of a sample for SEM visualization followed the protocol of Kaewkla and Franco (2019).

### Preliminary screening for antimicrobial activity against bacteria and fungi

The antimicrobial activity of strain TML10^T^ was tested on HPDA using a cross-streak method described previously (Williston et al., 1947; Kampapongsa and Kaewkla, 2016). There were three strains of Gram postivie bacteria: *Bacillus cereus* ATCC 11778, *Staphylococcus aureus* ATCC 25923 and Methicillin Resistant *S. aureus* (MRSA) DMST 20654; two strains of Gram-negative bacteria: *Pseudomonas aeruginosa* ATCC 27853 and a bacterial leaf blight pathogen, *Xanthomonas oryzae* PXO 71, and one yeast, *Candida albicans* BCC 7390. The antifungal assay by a dual culture method on HPDA followed a process described by Kampapongsa and Kaewkla (2016), and two fungi, *Curvularia lunata* BCC 15558 and *Fusarium incarnatum* BCC 4829, were used as test fungi. These tests were carried out in triplicate. ATCC strains were obtained from Department of Biology, Faculty of Science, Mahasarakham Univereity. MRSA DMST 20654 was obtained from the Department of Medical Sciences, Ministry of Public Health, Thailand. BCC strains were obtained from BIOTEC Culture Collection (BCC), Thailand, and *Xanthomonas oryzae* PXO 71 was obtained from Department of Medical Biotechnology, College of Medicine and Public Health, Flinders University, Australia.

### Liquid and solid-state fermentation of strain TML10^*T*^

Strain TML10^T^ was selected for small-scale liquid and solid-state fermentation. Strain TML10^T^ was cultured on HPDA for seven days, and two plugs of well-grown culture were inoculated into 50 ml of seed medium, IM22, in 250 ml baffled Erlenmeyer flasks, and incubated at 27°C, 150 rpm for 3 days. The composition of the seed medium_and four production liquid media, ISP 2, F26, F40, and SI (pH of all media was 7.4), is listed in Table 1. Seed culture (2.5 ml) was inoculated into 250 ml baffled Erlenmeyer flasks containing 50 ml of each production liquid medium and incubated at 27°C, 150 rpm in triplicate. One ml aliquot was collected daily for seven days and centrifuged at 5,000 rpm for 10 min. The supernatants were kept at −20°C until used. The mycelial pellets were extracted with 1 ml of absolute methanol on a shaker at 150 rpm, 25°C for 3 h. The methanol extract was centrifuged at 5,000 rpm for 10 min and then kept at −20°C for biological assay.

**Table 1:** Recipe of liquid and solid media for antibiotic production of strain TML10^T^.

**Media**	**Recipe (composition per 1 L RO water for broth media)**
IM 22 broth	Glucose 15 g, soyatone 15 g, pharmamedia 5 g, CaCO_3_ 2 g, NaCl 5 g
ISP 2 broth	Malt extract 10 g, glucose 4 g, yeast extract 4 g
F26 broth	Glucose 20 g, soy bean flour 10 g, CaCO_3_ 4 g, CoCl_2_.6H_2_O 1 mg
F40 broth	Glucose 0.5 g, soluble starch 15 g, malt extract 5 g, profolo 3 g, corn steep liquor 2 g, CaCO_3_ 2 g, MgSO_4_.7H_2_O 1 g, NaCl 2 g, trace elements solution 1 mL Trace elements solution was (composition per liter); CuSO_4_.5H_2_O 1 mg, FeSO_4_. 5H_2_O 7 mg, MnCl_2._4H_2_O 8 mg, ZnSO_4_.7H_2_O 2 mg
SI broth	Sucrose 20 g, CaCO_3_ 2.5 g, KNO_3_ 1 g, K_2_HPO_4_ 0.5 g, MgSO_4_.7H_2_O 0.5 g, NaCl 0.5 g
Solid medium	Basmati Rice 20 g, 5 ml of LF 42 medium containing 1 × HO-LE solution and 15 ml of RO water LF 42 medium; yeast extract 5 g, peptone 5 g, soya flour 5 g, glycerol 4 ml, soluble starch 2 g, CaCO_3_ 2 g, NaCl 2 g, K_2_HPO_4_ 0.5 g and MgSO_4_.7H_2_O 0.5 g/L RO water HO-LE solution (1,000 × ) contained (g/L) H_3_BO_3_ 2.85, MnCl_2_.4H_2_O 1.8, Sodium tartrate 1.77, FeSO_4_.7H_2_O 1.36, CoCl_2_.6H_2_O 0.04, CuCl_2_.2H_2_O 0.027, Na_2_MoO_4_.2H_2_O 0.025 and ZnCl_2_ 0.020

For solid-state fermentation, basmati rice was used (Table 1) in a 250 ml conical flask, as described by Kaewkla and Franco (2021). Briefly, rice medium with seed culture of strain TML10^T^ grown on IM22 prepared above was cultured for 7 days. About 1 g of rice medium from each flask was collected daily for seven days in triplicate. Each sample was extracted by using absolute methanol and shaken for 1 h. The crude extract of rice medium was centrifuged and kept at −20°C for biological assay. The purpose of extracting mycelial pellets and rice medium with absolute methanol was to extract polar compounds.

### Antibacterial and antifungal activity assays of strain TML10^*T*^

Fifty microliters of supernatants and methanol extract from pellets of all liquid cultured media and crude extract from a rice medium were tested against bacteria and yeast using an agar diffusion method (Kaewkla and Franco, 2021). Bacteria and yeast used for antimicrobial bioassay of antibiotic production by the agar diffusion method differed from the dual culture assay as this study was conducted in Australia. There was an antibiotic-resistant strain, *Pseudomonas aeruginosa* 06348315, and a sensitive strain of *Escherichia coli* JCM 109, for Gram-negative bacteria, a sensitive *S. aureus* ATCC 29213 and a methicillin-resistant *S. aureus* (MRSA) 03120385 for Gram-positive bacteria. A resistant *Candida albicans* ATCC 10231 was used for the anti-yeast assay. These tested microorganisms were obtained from the Clinical Microbiology Department, Flinders University, South Australia. Vancomycin (500 μg/ml), colistin (500 μg/ml), and amphotericin B (250 μg/ml) were used as standard antibiotics against Gram-positive bacteria, Gram-negative bacteria, and yeast, respectively. Absolute methanol and sterilized liquid media were used as negative controls. The test plates with tested bacteria and yeast were incubated at 37 and 27°C, respectively, for 18–24 h and were carried out in triplicate.

### Antibiotic production of strain TML10^*T*^

Based on the bioactivity, liquid medium F26 and a solid-state fermentation with a rice medium were tested for a large-scale antibiotic production of strain TML10^T^. Strain TML10^T^ was cultured in 650 ml of F26 in 3-L conical flasks in triplicate. Rice medium was prepared in 250 ml conical flasks in triplicate. Samples of culture broth and rice medium were collected daily for 6 days, and a bioassay was performed against *S. aureus* ATCC 29213 to assess the consistent production of the antibiotic. One ml of strain TML10^T^ supernatant cultured on F26 medium on day 2 and day 4 was freeze-dried, and its powder was dissolved with 100 μl of DI water, 30%, and 50% methanol. These solutions and a 100 μl of the rice extract in methanol were spotted on Merck Silica gel F254 TLC plate and run in an enclosed TLC tank containing system 1: chloroform: methanol (9:1). Bioautography of the active compounds was conducted according to Narasimhachari and Ramachandran (1967) against *S. aureus* ATCC 29213 and MRSA 03120385. The result showed that the crude extract from rice medium contained two compounds with antimicrobial activity: compound A (CA) (RF 0.69) and compound B (CB) (RF 0.53) in system 1. However, the concentrated compound from the F26 liquid medium collected on days 2 and 4 showed no activity (Supplementary Figure S1).

Therefore, the rice medium was selected for large-scale antibiotic production to purify the antibiotic and its structural elucidation as the production of bioactive compounds was stable compared to the liquid medium F26. Strain TML10^T^ was cultured in thirty-five 250 ml flasks of rice medium. After 3 days of growth, 60 ml of absolute methanol was added to each flask and shaken at 150 rpm at 25°C for 3 h. Methanol extracts were pooled and evaporated using a rotary evaporator and dried using a freeze-dryer.

### Purification of the antibiotic compounds

Four gram dried crude methanol extract of the culture in rice medium was dissolved with 70% aqueous methanol, and 3 g of silica gel 60 (200–400 mesh) was added. The solvents in this mixture were evaporated, and the powder was dried using a freeze-dryer. Silica gel (200 g; 200–400 mesh) was suspended in 100% chloroform and packed in a glass column. The freeze-dried crude compounds with silica gel 60 (prepared as above) were loaded into the column. The column was first eluted with 100% chloroform, and then a gradient of chloroform: methanol increasing from 0 to 5% methanol was used to elute the compounds. All fractions (68 fractions; 20 ml each) were tested for bioactivity against *S. aureus* ATCC 29213 and MRSA 03120385. Fractions 4–20 containing antibacterial activity were evaporated by an evaporator and dried using a freeze drier. Concentrated fractions 4–20 were spotted onto a TLC plate and run in an enclosed TLC tank containing system 1. Bioautography of the active compounds on a TLC plate was conducted against *S. aureus* ATCC, MRSA 03120385 (Supplementary Figure S2). Fractions 4–8 included the bioactive compound CA. Fractions 9–11 contained a mixture of CA and CB, while fractions 12–16 showed major compound CB (Supplementary Figure S3). The pooled fractions 4–8 and 9–20 were called pooled fractions (PFs) 1 and 2, respectively. These PFs were concentrated by an evaporator, and further purified by a preparative TLC with the same solvent system by spotting the purified compounds along the 16 cm length on the TLC sheet. The PF 1 contained only bands of CA, and the PF 2 included CA and CB; CA and CB were scraped from a silica gel sheet, eluted with 3 ml of chloroform: methanol (95:5), and evaporated to dryness. These purified compounds, CA and CB, were again spotted on a silica gel sheet to detect the pure compounds as single bands in the same elution system.

### High performance liquid chromatography

The purified antibiotics obtained by a preparative TLC, CA and CB, were analyzed by high performance liquid chromatography (HPLC) using ZORBAX Eclipse XDB-C18 column, 4.6 × 150 mm, 5 μm (Agilent Part No. 993967-902). The solvent system was acetonitrile: water with 0.1% trifluoroacetic acid by increasing gradient of 10% acetonitrile to 90% acetonitrile within 40 mins at a flow rate of 1.0 ml/min at 27°C. All samples were repletely injected three times.

### Structural elucidation by using liquid chromatography mass spectroscopy and nuclear magnetic resonance spectroscopy

The CA and CB analyzed by HPLC were purified compounds with retention times of 28.15 and 23.3 min, respectively (Supplementary Figure S4). Then, CA and CB were analyzed by reverse phase liquid chromatography-mass spectrometry (LC-MS) employing UV detection and electrospray mass spectrometry (ESI). The column, mobile phase, and LC condition were identical to the HPLC analysis. Based on LC-MS results of CA and CB, they had the same molecular weight of known antibiotics, naphthomycin A and B, respectively. CA and CB were run for H1 nuclear magnetic resonance (NMR), and only CA was analyzed for C13 NMR compared to the condition as previously described (Williams, 1975).

### Secondary metabolite and biosynthesis gene cluster prediction

Secondary metabolite analysis Shell (anti-SMASH) version 7.0 (Blin et al., 2023) was used to predict biosynthetic gene clusters (BGCs) of strain TML10^T^. Furthermore, the genome of strain TML10^T^ was examined by an *in silico* approach to search genes encoding metabolite products relating to antibiotic and bioactive compound production, plant growth promoting, and degradable enzymes. The UniProt database applying blastp (UniProt Consortium, 2023) was used to search for the closest similarity of microorganisms.

### Preliminary screening of arginine deiminase production of strain TML10^*T*^

Strain TML10^T^ was tested for arginine deiminase production by point-inoculating their cells on an M9 medium (pH 6.8 at 27°C) according to the protocol of Sharma et al. (2017). M9 medium with and without NaNO_3_ (5 g/L) instead of *L*-arginine was used as a negative control. The pinky-purple zone around bacterial cells showed a positive result.

### Protein sequences of arginine deiminase analysis and phylogenetic evaluation

Protein sequence of arginine deiminase of strain TML10^T^ was extracted from its genome and was BLAST-searched using Blastp on the UniProtKB database (UniProt Consortium, 2023).

The protein sequence of strain TML10^T^ was subsequently aligned with the representatives of the most closely related *Streptomyces* strains using CLUSTAL X (Thompson et al., 1997) with protein of *Bacillus licheniformis* as the outgroup. The protein alignment of all strains specified above was studied to find conserved and variable amino acids of strain TML10^T^ with other *Streptomyces* strains. The phylogenetic tree of the protein was constructed by the maximum likelihood (ML) algorithms (Saitou and Nei, 1987) using the software package MEGA version 11 (Tamura et al., 2021). The evolutionary distances of protein were computed using the Jones-Taylor-Thornton (JTT) parameter method (Jones et al., 1992). The tree's topology was evaluated using a bootstrap analysis based on 1,000 replications (Felsenstein, 1985).

### Statistical analysis

Antibiotic production of strain TML10^T^ in different media against tested microorganisms was carried out in triplicates, and inhibition zones were expressed as mean ± SD. IBM SPSS Statistics version 29 (Trial version software) was used to statistically analyze the different inhibition zone measurements. The normality of the data was tested by the Shapiro–Wilk test, and the results showed that the data significantly deviated from a normal distribution. Therefore, a non-parametric test with Kruskal–Wallis One-Way Analysis of Variance (ANOVA) was adopted to determine a significant group. Differences between means were considered significant at *p*-value < 0.05.

## Results and discussion

### Isolation of strain TML10^*T*^

*Streptomyces* sp. TML10^T^ was isolated from the surface-sterilized leaf of a Thai medicinal plant (*T. mucronata* Craib and Hutch). Dark green spores emerged on the leaf tissue placed on VL70 plus amino acid mixture medium (Schoenborn et al., 2004) after incubation for 9 weeks.

### Polyphasic study of strain TML10^*T*^

#### Genome *de novo* assembly of strain TML10^*T*^

The draft genome size of strain TML10^T^ sequenced by a long-read technique was 10.16 Mbp, containing 8,570 genes, with a DNA G + C content of 72.4 mol%. The draft genome of strain TML10^T^ contained (>1,000 bp) 14 contigs with the largest contig of 2.03 Mbp, and N50 was 1.23 Mbp. BUSCO result of draft genome *de novo* assembly of strain TML10^T^ was 99.9%. Although the genome assembly by long read techniques could not get a complete genome with 1–2 contigs, the result showed that 99.9% complete BUSCO compared with other strains in order Streptomycetales (1,579 genes). The genome of strain TML10^T^ and the genomes of five closely related type strains were checked for completeness and contamination using CheckM (Parks et al., 2015) and reported in Table 2. The completeness and contamination of the genome of strain TML10 were 99.53 and 1.33%, respectively.

**Table 2:** 16S rRNA gene sequence similarity, average nucleotide identities, digital DNA:DNA hybridization values (%) between strain TML10^T^ and their related species.

**Strain/comparison with 1**.	**2**.	**3**.	**4**.	**5**.
16S rRNA gene similarity (%)	97.1	97.2	98.9	99.2
(ANIb) (%)	88.5	88.5	87.2	84.1
(ANIm) (%)	90.8	90.7	90.4	88.4
dDDH (%)	38.8 (C.I. model 36.4–41.4)	38.6 (C.I. model 36.1–41.3)	37.8 (C.I. model 35.3–40.3)	31.8 (C.I. model 29.4–34.3)
Genome completeness and contamination (%)	100, 1.9	99.86, 0.82	99.53, 1.33	99.47, 2.62

Strain: 1, *Streptomyces naphthomycinicus* TML10^T^; 2, *Streptomyces musisoli* CH5-8^T^; 3, *Streptomyces echinatus* CECT 3313^T^; 4, *Streptomyces corchorusii* DSM 40340^T^; 5, *Streptomyces cellostaticus* NBRC 12849T.

### 16s rRNA gene and phylogenetic analysis of strain TML10^*T*^

The 16S rRNA gene length of strain TML10^T^ was 1,453 bp. Four type strains shared the highest 16S rRNA gene sequence similarity with strain TML10^T^ at ≥99.0%: *S. cellostaticus* NBRC 12849^T^ (99.2%), *Streptomyces yokosukanensis* NRRL B-3353^T^ (99.0%), *Streptomyces bungoensis* DSM 41781^T^ (99.0%), and *Streptomyces cinnabarigriseus* JS360^T^ (99.0%). The 16S rRNA gene phylogenetic evaluation showed that strain TML10^T^ was encompassed with other members of *Streptomyces*. The maximum likelihood tree showed that the closest neighbor of strain TML10^T^ was *Streptomyces variegatus* NRRL B-16380^T^ (98.9% 16S rRNA gene similarity). The two closest type strains that shared the highest dDDH values, *S. musisoli* CH5-8^T^ and *Streptomyces echinatus* JCM 4144^T^, positioned in a different cluster were well separated from strain TML10^T^ on the ML tree (Supplementary Figure S5).

### Genome comparison study

The two closest type strains which shared the highest dDDH, ANIb, and ANIm values with strain TML10^T^ were *S. musisoli* CH5-8^T^, and *S. echinatus* JCM 4144^T^, at 38.8%, 88.5%, 90.8%; and 38.6%, 88.5%, 90.7%, respectively (Table 2). However, these two type strains shared very low 16S rRNA gene similarity with strain TML10^T^ at 97.1, and 97.2%, respectively. On the other hand, these values between strain TML10^T^ and *S. cellostaticus* NBRC 12849^T^, which shared the highest 16S rRNA gene similarity (99.2%), were 31.8%, 84.1%, and 88.4%, respectively.

**Figure 1:**
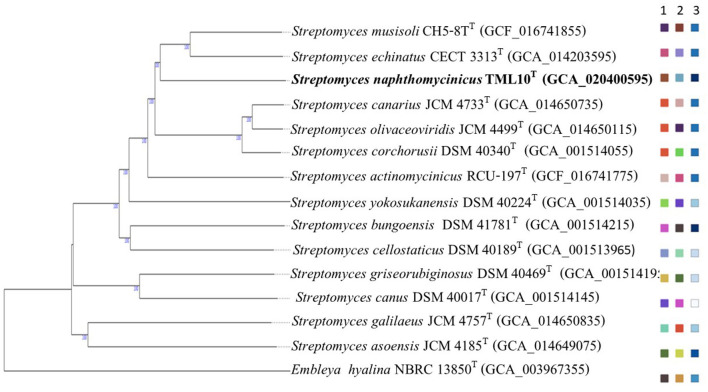
Phylogenomic tree based on TYGS result showing relationship between *Streptomyces naphthomycinicus* TML10^T^ with related type strains. The numbers above branches are GBDP pseudo-bootstrap support values >60% from 100 replications, with an average branch support of 97.5%. The tree was rooted at the midpoint (Farris, 1972). Leaf labels are annotated by affiliation to species (1) and subspecies clusters (2) and genomic G+C content (3) (Meier-Kolthoff and Göker, 2019).

The phylogenomic tree of strain TML10^T^ and the closely related type strains showed that strain TML10^T^ forms a different cluster with the closest neighbor, *S. musisoli* CH5-8^T^, and *S. echinatus* ISP 5013^T^ with different species cluster (Figure 1). Similarly, the ML phylogenomic tree using the codon tree option in the PATRIC web server showed that these type strains were the closest neighbors of strain TML10^T^ but formed the different cluster (Supplementary Figure S6). It was reported that the species delineation should have ANI value lower than 95%−96% (Richter and Rosselló-Móra, 2009) and dDDH value was lower than the threshold of 70% used to define species level (Meier-Kolthoff et al., 2013; Chun et al., 2018). Then, the three closest related species, *S. musisoli* CH5-8^T^, *S. echinatus* CECT 3313^T^, *S. corchorusii* DSM 40340^T^, *S. cellostaticus* NBRC 12849^T^ were selected for physiological and biochemical comparison.

### Chemotaxonomic, cultural and morphological properties of strain TML10^*T*^

Strain TML10^T^ was found to have chemotaxonomic, cultural, and morphological properties consistent with its classification in the genus *Streptomyces* (Kämpfer, 2012). Colonies were tough with white or grayish-green spores. Substrate and aerial mycelia of strain TML10^T^ developed well in most media used. This strain produces grayish-green spores on ISP 2, ISP 4, ISP7, and Bennet's agar. Melanin pigment was produced on ISP 7. Strain TML10^T^ had loop spores with spiny surfaces. The morphology of strain TML10^T^ is described in Supplementary Table S1. Electron micrographs revealed that it formed spores in spiral chains (~1.0 × 1.0 microns) with spiny surfaces (Supplementary Figure S7). Whole cells contain *LL*-diaminopimelic acid in its peptidoglycan and galactose, glucose, and mannose as whole-cell sugars. Major lipids were phosphatidylethanolamide (PE), phosphatidylglycerol (PG), phosphatidylinositol (PI), an unknown lipid with amino group (LPA), and an unknown lipid with phosphate group (LPL), which corresponded to phospholipid type I (Lechevalier et al., 1977) (Supplementary Figure S8). MK-9(H_6_) and MK-9(H_8_) are the predominant menaquinones. The major cellular fatty acids of this strain (≥10%) were *anteiso*-C_15:0_ (23.8%), *anteiso*-C_17:0_ (20.1%), and *iso*-C_16:0_ (15.3%) (Supplementary Table S2). The major fatty acids of the closest type strain, *S. musisoli* CH5-8^T^ (≥10%), were *anteiso*-C_15:0_ (25.6%), *iso*-C_16:0_ (22.0%), and *anteiso*-C_17:0_ (17.1%). This type strain was cultured on ISP 2 liquid medium by shaking at 200 rpm at 30°C for 5 days (Duangupama et al., 2021).

### Phenotypic and physiological characterization

The physiological properties that differentiate strain TML10^T^ from four closely related species are shown on the Table 3. The aerial mass color of strain TML10^T^ on ISP 2 differed from that of other type strains. Additionally, strain TML10^T^ could not produce diffusion pigment on ISP2, while the closest type strain, *S. musisoli* CH5-8^T^, could produce pigment. The characteristics of strain TML10^T^ in terms of growth at pH 5, maximum NaCl tolerance (w/v), and maximum growth temperature were distinct from those of the two closest type strains, *S. musisoli* CH5-8^T^ and *S. echinatus* CECT 3313^T^ (Table 3). Strain TML10^T^ could hydrolyze starch, but *S. musisoli* CH5-8^T^ and *S. echinatus* CECT 3313^T^ could not and weakly hydrolyzed it, respectively. The spore surface of strain TML10^T^ was spiny, while the spore surface of *S. musisoli* CH5-8^T^ was rough.

**Table 3:** Differential characteristics between *Streptomyces napthomycinicus* TML10^T^ and related species of *Streptomyces*.

**Characteristics/strains**	**1^#^**.	**2^a^**.	**3**.	**4**.	**5^#^**.
Spore chain	Loop	Spiral	Spiral^c^	Spiral^c^	Spiral^c^
Spore surface	Spiny	Rough	Spiny^c^	Smooth^c^	Spiny^c^
Aerial mass color on ISP 2	Grayish green	Medium gray	Gray^c^	Light grayish yellowish brown^c^	Pale yellowish green
Diffusion pigment on ISP2	–	+ (brilliant yellow)	–^c^	–^c^	–
Melanin pigment production on ISP 7	+	+	+^c^	–^c^	+
Growth at pH 4	–	–	–^a^	+^b^	–
Minimum pH for growth	5	6	6^a^	nd	5
Maximum NaCl tolerance (%, w/v)	5	7	3^a^	2 (w)^d^	5
Maximum temperature for growth (°C)	37	45	42^a^	nd	37
Hydrolyze of starch	+	–	w^a^	+^d^	+

Strain: 1, *Streptomyces napthomycinicus* TML10^T^; 2, *Streptomyces musisoli* CH5-8^T^; 3, Streptomyces echinatus JCM 4144^T^; 4, *Streptomyces corchorusii* NBRC 13032^T^, 5, *Streptomyces cellostaticus* NBRC 12849^T^. +, positive or present; –, negative or absent; w, weak; nd, no report. Data was taken from ^a^Duangupama et al. (2021); ^b^Také et al. (2015); ^c^Kämpfer (2012); ^d^Law et al. (2019); ^#^all data from this study.

Data from the literatures used the same methods with this study except; ^a^The pH range for growth was tested in ISP 2 broth. ^d^NaCl tolerance was tested in tryptic soy broth.

Based on the polyphasic taxonomy, strain TML10^T^ was proposed as a novel species named *Streptomyces naphthomycinicus* sp. nov.

### Antimicrobial assay and antibiotics production of strain TML10^*T*^

The result of a dual culture assay showed that strain TML10^T^ strongly inhibited *B. cereus* ATCC 11778, *C. albicans* BCC 7390, and *C. lunata* BCC 15558 and showed good activity against *S. aureus* ATCC 25923 and MRSA DMST 20654. The antimicrobial activity of strain TML10^T^ is shown in Table 4.

**Table 4:** Antimicrobial activity of strain TML10^T^ against tested microorganism by dual culture technique.

**Strain**	**Inhibition against**							
	***Bacillus*** **cereus ATCC 11778**	**MRSA DMST 20654**	***Staphylococcus*** **aureus ATCC 25923**	***Xanthomonas*** **oryzae PXO 71**	***Pseudomonas*** **aeruginosa ATCC 27853**	***Candida*** **albicans BCC 7390**	***Curvularia*** **lunata BCC 15558**	***Fusarium*** **incarnatum BCC 4829**
TML10^T^	++++	+++	+++	++	++	++++	++++	++

++++, strong inhibition; +++, good inhibition; ++, moderate inhibition; +, weak inhibition.

The antimicrobial activity of strain TML10^T^ on four liquid media and a solid medium with rice grain by an agar diffusion method is shown in Table 5. The result showed that strain TML10^T^ on F26 liquid medium was potent inhibition against *S. aureus* ATCC 29213 with a clear zone of 14.3 ± 0.28 mm. Supernatants from F26 and F40 media showed moderate inhibition against MRSA 03120385 (9.7 ± 0.28 and 10.2 ± 0.28 mm) and *C. albicans* ATCC 10231 (8.3 ± 0.28 and 8.2 ± 0.28 mm). Bioactive compounds from these liquid media were derived from the supernatant but not the mycelium extracted with methanol. The solid medium with rice grain was the best to produce antibiotics against *S. aureus* ATCC 29213, MRSA 03120385, and *C. albicans* ATCC 10231 (17.7 ± 0.28, 18.3 ± 0.57, and 12.8 ± 0.28 mm), comparable to the antibiotic standard (18.8 ± 028, 18.7 ± 0.57, and 14.5 ± 0.5 mm) with not significantly difference at *p*-value < 0.05 (Table 5).

**Table 5:** Antibiotics production of strain TML10^T^ in four liquid media and rice medium against test organisms by agar diffusion assay.

**Medium**	**Inhibition zone (mm)** ^**#**^				
	***Staphylococcus*** **aureus ATCC 29213**	**MRSA 03120385**	***Escherichia*** **coli JCM 109**	***Pseudomonas*** **aeruginosa 06348315**	***Candida*** **albicansATCC 10231**
ISP 2	–	–	–	–	–
Met+ISP2	–	–	–	–	–
F 26	14.3 ± 0.28ab	9.7 ± 0.28a	–	–	8.3 ± 0.28ab
Met+F26	–	–	–	–	–
F40	12.6 ± 0.12a	10.2 ± 0.28ab	–	–	8.2 ± 0.28a
Met+F40	–	–	–	–	–
S1	–	–	–	–	–
Met+S1	–	–	–	–	–
Rice medium	17.7 ± 0.28ab	18.3 ± 0.57ab	–	–	12.8 ± 0.28ab
Vancomycin 500 μg/ml	18.8 ± 028b	18.7 ± 0.57b	nd	nd	nd
Colistin 500 μg/ml	nd	nd	17 ± 0.28	11.5 ± 0.25	nd
Amphotericin B 250 μg/ml	nd	nd	nd	nd	14.5 ± 0.5b

^#^Values are mean of triplicate determination (*n* = 3) ± standard deviation.

–, no zone of inhibition was found; Met+, methanol extracted from cells which grown on each medium; nd, not done. Lower case letters in the same column indicate significant differences (p < 0.05).

### Antibiotic production in F26 liquid medium and a rice medium of strain TML10^*T*^

**Figure 2:**
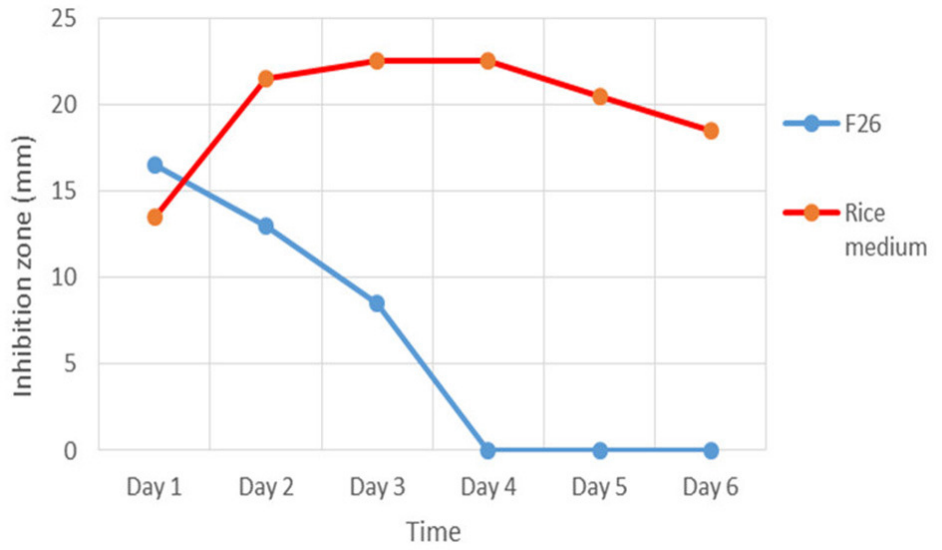
Antibiotic production of strain TML10^T^ in F26 liquid medium and solid state with rice grain inhibit *Staphylococcus aureus* ATCC 29213 for 6 days.

The results showed that the maximum antibiotic production of strain TML10^T^ against *S. aureus* ATCC 29213 in F26 liquid medium was at day 1 (16.5 mm) and was dramatically decreased to zero at day 4 (Figure 2). In contrast, antibiotic production in a rice medium gradually increased from day 1 and yielded the maximum production at day 3. The TLC result showed that the concentrated supernatant of the F26 medium on days 2 and 4 showed fewer compounds than the crude extract of the rice medium's TLC profile (Supplementary Figure S1). In this study, solid-state fermentation was applied successfully for antibiotic production. Using solid-state fermentation would be beneficial in terms of consistency in production, requiring less energy, and lowering cost by using a variety of agricultural wastes (Robinson et al., 2001). For example, a solid state with corncob was used for oxytetracycline production by *Streptomyces rimosus* TM-55 (Yang, 1996). Many solid ferments with rice grain were successfully used for antibiotics production (Ellaiah et al., 2002, 2004; Saykhedkar and Singhal, 2004; Nagavalli et al., 2015). Based on this study, solid fermentation was better than liquid fermentation for a bioactive compound screening program.

### Structural elucidation of compound A and B by LC-MS and NMR analysis

Molecular weight of compound A (CA) was (M + H) = 718; (M + Na) = 742 and compound B (CB) was (M + H) = 706; (M + Na) = 728 (Supplementary Figure S9). Based on these molecular weights and H^1^ NMR (Supplementary Figure S10), CA and CB corresponded to known compounds, naphthomycin A (C_40_H_46_NO_9_Cl) and naphthomycin B (C_39_H_44_NO_9_Cl), respectively. Only CA was selected to do C^13^ NMR. Along with these H^1^ NMR and C^13^ NMR results, compound A was confirmed as naphthomycin A (Supplementary Figure S10) (Williams, 1975). CB was not further analyzed; it has a high possibility of being naphthomycin B or its isomer, naphthomycin H (Mukhopadhyay et al., 1985). Naphthomycin and its derivative are the antibiotics produced by many strains of *Streptomyces*. It belongs to ansamycin antibiotics, which showed antibacterial, antifungal, and antitumor activity (Mukhopadhyay et al., 1985). Recently, 17 derivatives of naphthomycin have been reported, and some derivatives showed only antitumor activity against human cancer (Zhang et al., 2019).

### Biosynthesis gene cluster prediction and *in silico* gene prediction of the genome of strain TML10^*T*^

There were six types of BGCs (≥50% similarity) detected from a draft genome of strain TML10^T^ (Table 6). Of the terpenes, carotenoid (63%), geosmin (100%), and hopene (92%) were compounds commonly found in *Streptomyces* spp., and albaflavenone (100%), a tricyclic sesquiterpene antibiotic, was reported with antibacterial activity produced by *Streptomyces* (Moody et al., 2011). Type I PKS included a macrocyclic lactam antibiotic, tripartilactam (100%), and naphthomycin A (71%), which was in correlation with this study that strain TML10^T^ produced naphthomycin A and its derivative. Strain TML10^T^ contains a lantipeptide BGC that showed 58% similarity to the reported informatipeptin BGC. The NRPS cluster contains rimosamide (50%), exhibiting antibiotic activity (McClure et al., 2016). BGCs of other compounds, including melanin (71%), ectoine (100%), and desferrioxamin B/ E (83%), were detected in the genome of strain TML10^T^. Based on the phenotypic study of strain TML10^T^, this strain produced melanin pigment on ISP 7 medium, which correlated with its genome data. Melanin has the potential to be used in various fields, such as antitumor, scavenger of free radicals, antimicrobial, neuroprotector, antivenin stimulator, liver protector, anti-inflammatory, and protector of the digestive system (El-Naggar and Saber, 2022). Moreover, strain TML10^T^ contained BGC of ε-Poly-L-lysine (ε-PL) (100%), a microbial peptide that applies to use for preserving packaged food. ε-PL is widely used worldwide due to its broad antimicrobial activity against Gram-negative and Gram-positive bacteria, yeasts, and molds (Ye et al., 2013). Wang et al. (2021) reported that the genome mining technique was a high-throughput screening for strains that carry ε-PL synthetase (*pls* gene). The strain that produces a high yield of ε-Poly-L-lysine, *Streptomyces albulus* NK660, was thoroughly studied Pls protein (*pls* gene) and revealed that *pls* gene sequences and its homologs in Streptomycetes are well conserved (Geng et al., 2014). Moreover, a gene encoding a Pls-like protein was detected in the genome of *Corynebacterium variabile* with the similarity between this protein and Pls from *S. albulus* is only 51%; they share highly similar domain architecture (Jiang et al., 2021). However, the conditions when this BGC is active (if ever) in these particular strains are not known. Then, strain TML10^T^, which shared 100% gene similarity with known BGC of ε-PL, is likely to produce this compound and be a potent candidate as an antibiotic producer for commercial scale in the future. Although AntiSMASH is an effective tool for finding similar known BGCs, it is limited to annotating peptides and polyketides coded by modular assembly lines only. Moreover, the annotation of chemical compounds coded by cyclization and tailoring reactions is still limited. A multiple possible end-product compound strategy can be applied to overcome this limitation (Weber et al., 2015). Further experiments by applying suitable media for ε-PL production will be required for ε-PL production in the future.

**Table 6:** The distribution of BGCs of *Streptomyces naphthomycinicus* TML10^T^ based on “antiSMASH” prediction.

**BGC type**	**Product**	**Span (nt)**	**BGC similarity (%)**
Terpene	Albaflavenone	58,820–79,833	100
	Carotenoid	1,598,386–1,623,976	63
	Geosmin	928,798–950,957	100
	Hopene	309,016–334,735	92
Type 1 PKS (T1PKS)	Tripartilactam/niizalactam	1,408,322–1,541,131	100
	Naphthomycin A	1,387,838–1,498,759	71
Type 2 PKS (T2PKS)	Spore pigment	644,852–717,363	83
NRPS	Rimosamide	190,892–234,867	42
	ε-Poly-L-lysine	779,032–812,910	100
RiPP: Lanthipeptide	Informatipeptin	874,756–954,119	57
Others	Melanin	1,068,663–1,089,031	71
	Ectoine	549,991–559,379	100
	Desferrioxamin B/desferrioxamine E	840,565–852,334	83

The *in silico* gene prediction results showed that strain TML10^T^ contained various genes encoding antibiotic production: actinorhodin, gramicidin, tyrocidine, and an antitumor compound, chondramide (Supplementary Table S3). Germicidin is an auto-regulative inhibitor of spore germination in the genus *Streptomyces* (Petersen et al., 1993). This strain contains genes encoding drought tolerance and stress response proteins such as ectoine production, glycine betaine/L-proline transporter, glycine, betaine and proline production, and sodium solute symporter (Horn et al., 2006). In correlation with the ecophysiology, the Phujongnayoi National Park, where the host plant of strain TML10^T^ is located, is an arid area for 7 months in cold and summer seasons. Strain TML10^T^ might help plants survive a dry season. The genome of strain TML10^T^ contains many genes encoding beneficial enzymes that can be applied in numerous industries, such as alpha-N-arabinofuranosidase, amylase, xylosidase, and β-xylanase (Podkaminer et al., 2012). Strain TML10^T^ contained a gene encoding arginine deiminase, an invaluable enzyme for anticancer (Maghnouj et al., 1998).

### Arginine deiminase production, phylogenetic tree and protein alignment of arginine deiminase protein of strain TML10^*T*^

Strain TML10^T^ could degrade arginine and use it as a nitrogen source as they gave positive results in producing a pink purple color around their colony on M9 agar.

**Figure 3:**
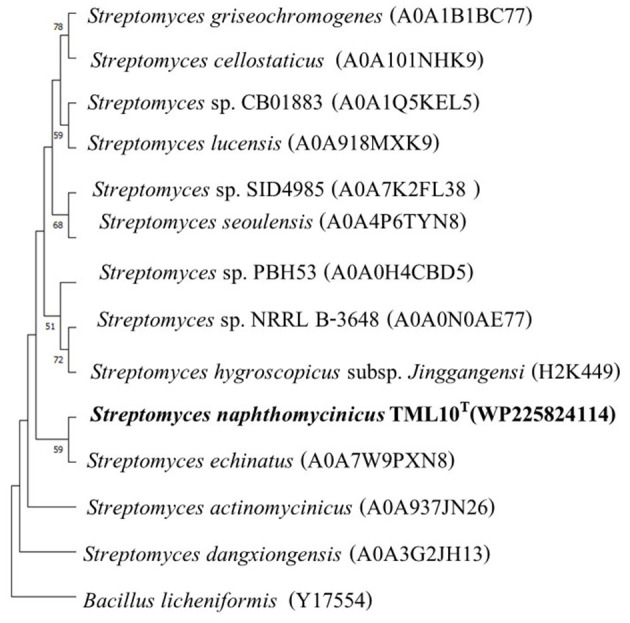
Maximum-likelihood phylogenetic tree based on protein of arginine deiminase sequences of strains TML10^T^ and its nearest phylogenetic neighbors in genus *Streptomyces* and *Bacillus licheniformis* as the out-group (414 bp). Bootstrap values based on 1,000 replicates are shown at the branch nodes. The scale bar represents 0.010 changes per nucleotide.

The result of the blast search using Blastp showed that the closest protein of strain TML10^T^ was *Streptomyces echinatus*, which shared 97.3% similarity, and strain TML10^T^ shared 93.9–96.1% similarity with 11 strains of *Streptomyces* sp. The arginine deiminase protein of strain TML10^T^ shared only 40% similarity with *B. licheniformis*. The protein alignment showed that protein sequences of strain TML10^T^ and all *Streptomyces* strains, including *B. licheniformis* contained conserved amino acids (red color; Supplementary Table S4), which are known to be essential for the full enzymatic activity of the protein (Das et al., 2004). Moreover, three amino acids of strain TML10^T^ differed from other *Streptomyces* (green color). This difference indicated the gene mutation of strain TML10^T^, which might affect the protein function or enzyme property (Maghnouj et al., 1998). The ML phylogenetic tree showed that the protein of *S. echinatus* (97.3% similarity) was the closest neighbor of strain TML10^T^, which was positioned in the same clade (Figure 3). *B. licheniformis* ATCC 14580 was reported to produce arginine deiminase and contains clustered in an operon-like structure in the order *arc*A (arginine deiminase), *arc*B (ornithine carbamoyltransferase), *arc*D (putative arginine-ornithine antiporter) and *arc*C (carbamate kinase) genes. *Arc*A, *arc*B, and *arc*C were reported as the crucial genes for the arginine deiminase pathway of many bacteria (Maghnouj et al., 1998). Gene encoding ornithine carbamoyltransferase is detected in strain TML10^T^ genome. However, genes encoding carbamate kinase could not be detected in the genomes of strain TML10^T^. Based on the protein arginine deiminase alignment of strain TML10^T^ with *B. licheniformis*, the protein sequence of strain TML10^T^ contained all conserved amino acids, which are essential for the full enzymatic activity of the protein. Then, strain TML10^T^ and closely related *Streptomyces* in this protein alignment study should present enzyme arginine deiminase activity. It is worth studying the pathway of arginine deiminase of strain TML10^T^ in depth to determine if it could be a potent candidate for arginine deiminase production in the future. In addition, the production of arginine deiminase by strain TML10^T^ in liquid culture and analysis of arginine deiminase activity are required to prove whether this strain can produce arginine deiminase, which is anti-cancer worldwide.

### Description *of Streptomyces naphthomycinicus* sp. nov.

*Streptomyces naphthomycinicus* (*naph.tho.my.ci'ni.cus*. N.L. masc. adj. *naphthomycinicus*, belonging to the antibiotic naphthomycin).

Aerobic, catalase positive. Cells grow between 15 and 37°C with good growth at 27°C. Cells grow between pH 5 and pH 10, with optimum growth at pH 7. Colonies are tough with white or grayish-green spores. Substrate and aerial mycelia develop well on most media used. Melanin pigments are produced on ISP 7. This strain produces grayish-green spores on ISP 2, ISP 4, ISP 7, and Bennet's agar. Strain TML10^T^ grows moderately on nutrient agar with white spores. The mycelium is extensively branched and forms loop spores (~1.0 × 1.0 μm) with spiny surfaces. Strain TML10^T^ produces acids from arabinose, cellobiose, fructose, galactose, glucose, maltose, mannose, *myo*-inositol, mannitol, raffinose, rhamnose, ribose, sorbitol, sucrose, salicin, sorbitol, trehalose, and xylose but not dulcitol. Cells hydrolyze starch and skim milk, *L*-tyrosine, adenine, and xanthine but not esculin, hippurate, and urea. Cells assimilate acetate, citrate, propionate, benzoate, and tartrate but not malate. Strain TML10^T^ cannot use pyridine, toluene, phenol, and benzene as the sole carbon source. Whole cells contain *LL*-diaminopimelic acid in its peptidoglycan and galactose, glucose, and mannose as whole-cell sugars. The type strain contains MK-9(H_6_) and MK-9(H_8_) as the predominant menaquinone. The major cellular fatty acids of the type strain (≥10%) were *anteiso*-C_15:0_, *anteiso*-C_17:0_, and *iso*-C_16:0_. Major phospholipids are phosphatidylethanolamide (PE), phosphatidylglycerol (PG), phosphatidylinositol (PI), an unknown lipid with amino group (LPA) and an unknown lipid with phosphate group (LPL). The draft genome size of strain TML10^T^ is 10.16 Mbp, and the DNA G+C content is 72.4 mol%. The type strain, TML10^T^ (=TBRC 15050^T^ = NRRL B-65638^T^), is an endophytic actinobacterium isolated from a leaf sample of a Thai medicinal plant (*Terminalia mucronata* Craib and Hutch) which grows in the Phujongnayoi National Park, Ubonratchathani province, Thailand. Strain TML10^T^ is capable of producing naphthomycin A and its derivative. The GenBank/EMBL/DDBJ accession number for the 16S rRNA gene sequence and whole genome sequence of strain TML10^T^ are MZ901364 and JAINRE000000000, respectively.

## Conclusion

A novel species of the genus *Streptomyces* named *S. naphthomycinicus*, TML10^T^, was discovered from the surface-sterilized leaf of a Thai medicinal plant. This strain is capable of producing naphthomycin A, an ansamycin antibiotic. The findings of this study provide valuable insights into selecting appropriate antibiotic production media, which is critical for a successful antibiotic screening program. Genomic data mining revealed a correlation between the genotypic and phenotypic characteristics of this strain, which contains BGCs of naphthomycin A and the *arc*A gene encoding arginine deiminase.

In addition, genome data mining based on antiSMASH and Thin-Layer Chromatography profiling of its extracted compounds suggest that strain TML10^T^ has the potential to produce valuable compounds such as ε-Poly-L-lysine. Further research is required to confirm whether this strain can produce arginine deiminase in liquid culture and contains the necessary enzymatic activity to be useful for arginine deiminase production, a treatment for cancer worldwide. Additionally, exploring various suitable production media under optimized conditions will benefit in discovering other valuable compounds from this strain.

## Data availability statement

The datasets presented in this study can be found in online repositories. The names of the repository/repositories and accession number(s) can be found in the article/Supplementary material.

## Author contributions

OK: Conceptualization, Data curation, Formal analysis, Investigation, Methodology, Project administration, Resources, Validation, Writing – original draft, Writing – review & editing. MP: Data curation, Formal analysis, Investigation, Validation, Writing – review & editing. AT: Supervision, Validation, Writing – review & editing. WS: Funding acquisition, Writing – review & editing. SS: Data curation, Formal analysis, Writing – review & editing. CS: Data curation, Formal analysis, Investigation, Writing – review & editing. NC: Formal analysis, Investigation, Writing – review & editing. TC: Formal analysis, Investigation, Validation, Writing – review & editing. CF: Methodology, Resources, Supervision, Validation, Writing – review & editing.
