# Supplementary material for: Description of Streptomyces naphthomycinicus sp. nov., an endophytic actinobacterium producing naphthomycin A and its genome insight for discovering bioactive compounds
Source: Front Microbiol. 2024 Apr 17;15:1353511. doi: 10.3389/fmicb.2024.1353511 (PMC11061393; doi:10.3389/fmicb.2024.1353511)
Supplement: Supplementary file 1 [file Data_Sheet_1.PDF]

Supplementary Figures

Description of *Streptomyces naphthomycinicus* sp. nov., an endophytic actinobacterium producing naphthomycin A and its genome insight for discovering bioactive compounds

Onuma Kaewkla[1,2*], Mike Perkins[3], Arinthip Thamchaipenet[4], Weerachai Saijuntha[1,5], Sudarat Sukpanoa[6], Chanwit Suriyachadkun[7], Nitcha Chamroensaksi[8], Theeraphan Chumroenphat[9], Christopher Milton Mathew Franco[2]

[1] Center of Excellence in Biodiversity Research, Mahasarakham University, Maha Sarakham 44150, Thailand
[2]Department of Medical Biotechnology, College of Medicine and Public Health, Flinders University, Adelaide, Australia
[3]Deparment of Chemistry, College of Science and Engineering, Flinders University, Adelaide, Australia
[4]Department of Genetics, Kasetsart University, Chatuchak, Bangkok 10900, Thailand
[5] Faculty of Medicine, Mahasarakham University, Maha Sarakham 44000, Thailand
[6]Department of Biology, Faculty of Science, Mahasarakham University, Maha Sarakham 44150, Thailand
[7]Thailand Bioresource Research Center (TBRC), National Center for Genetic Engineering and Biotechnology, National Science and Technology Development Agency, Klong Luang, Pathumthani 12120, Thailand
[8]National Biobank of Thailand (NBT), National Center for Genetic Engineering and Biotechnology, National Science and Technology Development Agency, Klong Luang, Pathumthani 12120, Thailand
[9]Aesthetic Sciences and Health Program, Faculty of Thai Traditional and Alternative Medicine, Ubon Ratchathani Rajabhat University, Ubon Ratchathani 34000, Thailand

* Corresponding author:
Onuma Kaewkla
Email address: Onuma.k@msu.ac.th

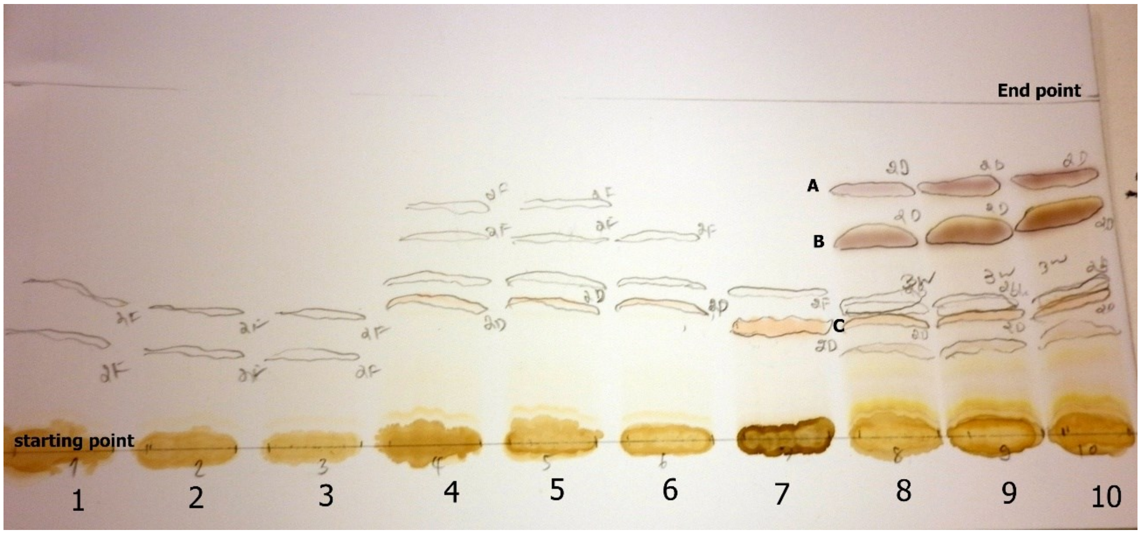

Supplementary figure S1. TLC profile of antibiotics production of strain TML10 on different medium (solvent system; chloroform: methanol (9:1)) A, compound A; B, compound B

Lane 1; Freeze dried broth of F26 harvest 2 d dissolved with water
Lane 2; Freeze dried broth of F26 harvest 2 d dissolved with 30% methanol
Lane 3; Freeze dried broth of F26 harvest 2 d dissolved with 50% methanol
Lane 4; Frozen dried broth of F26 harvest 4 d dissolved with water
Lane 5; Frozen dried broth of F26 harvest 4 d dissolved with 30% methanol
Lane 6; Frozen dried broth of F26 harvest 4 d dissolved with 50% methanol
Lane 7; Strain TML10 grown on ISP 2 agar and extracted with absolute methanol and dried compound dissolve with 50% methanol
Lane 8: Extracted rice medium dissolved with 30% methanol
Lane 9: Extracted rice medium dissolved with 50% methanol
Lane 10: Extracted rice medium dissolved with 70% methanol

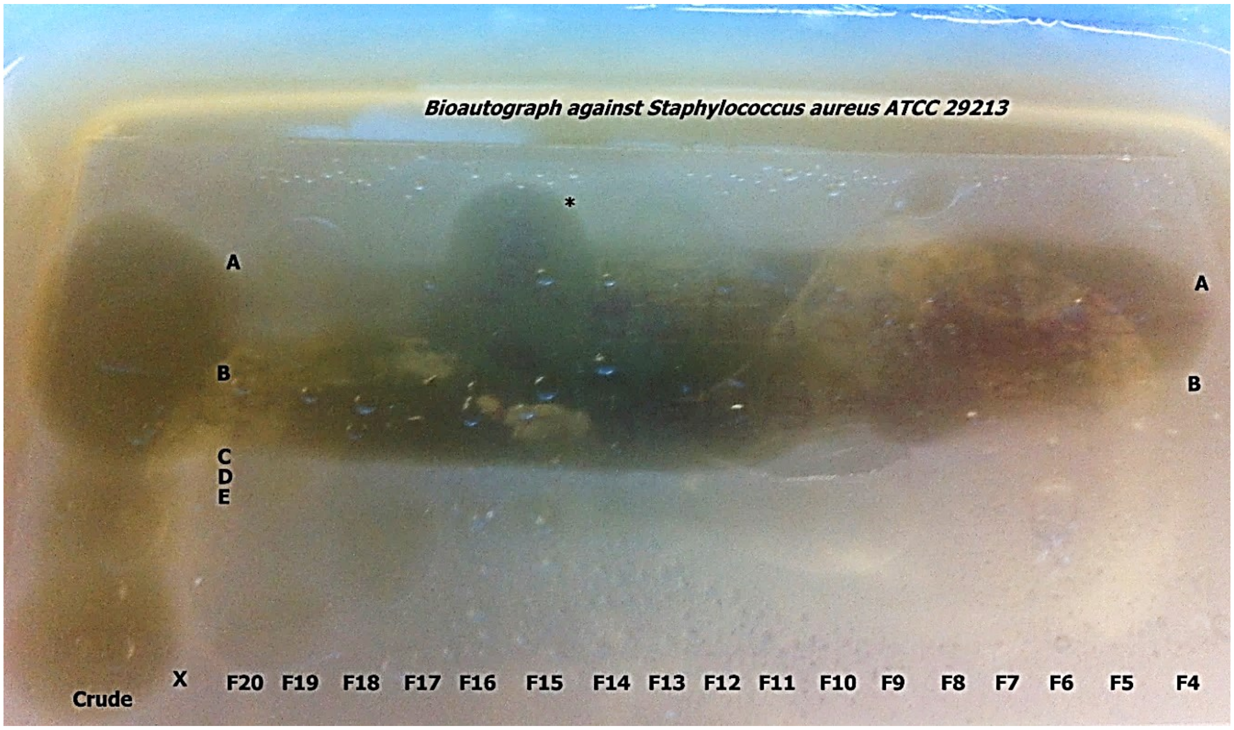
Bioautograph against Staphylococcus aureus ATCC 29213
*
A
B
C
D
E
A
B
Crude
X
F20 F19 F18 F17 F16 F15 F14 F13 F12 F11 F10 F9 F8 F7 F6 F5 F4

A)

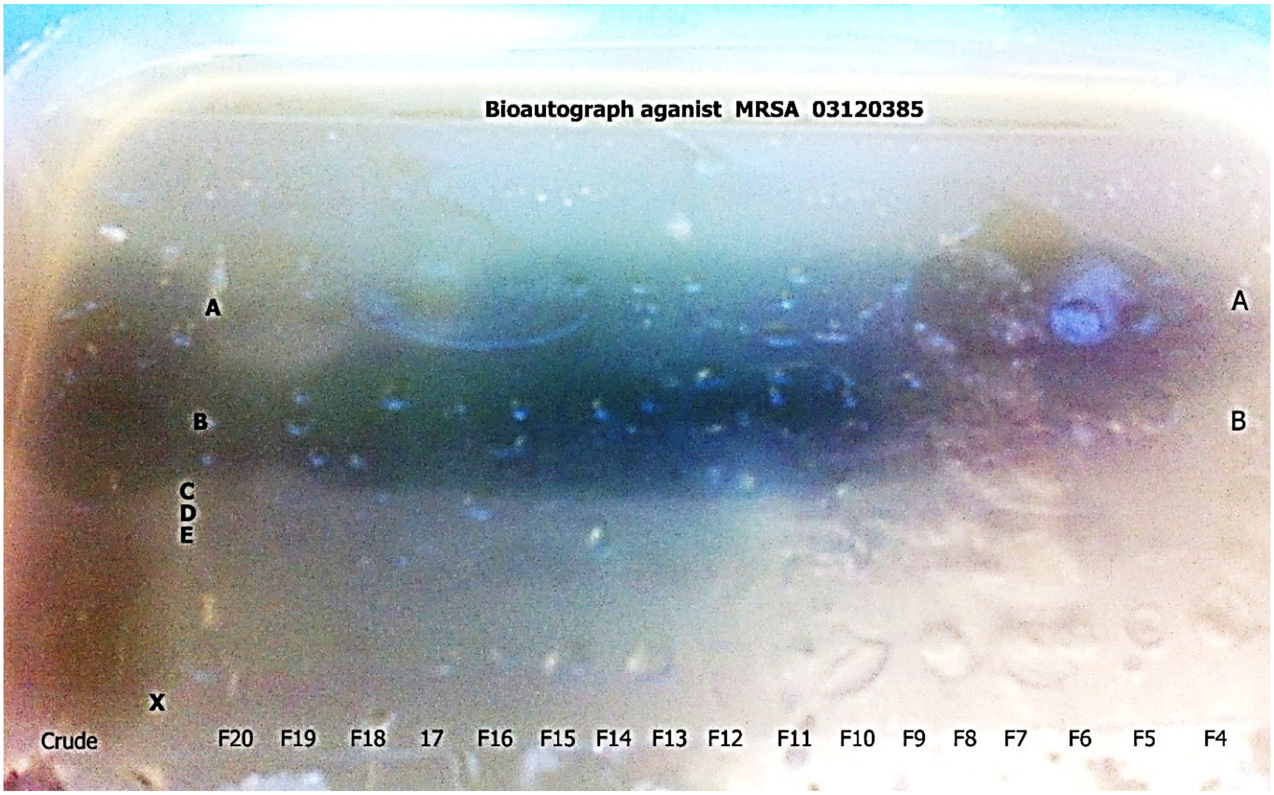
Bioautograph aganist MRSA 03120385
A
B
C
D
E
A
B
X
Crude
F20 F19 F18 17 F16 F15 F14 F13 F12 F11 F10 F9 F8 F7 F6 F5 F4

B)

Supplementary figure S2 Bioautogram on silicagel 60 F_{254} of fraction 4-20 from silica gel column of bioactive compounds of strain TML10^{T} against A) *S. aureus* ATCC 29213; B) MRSA 03120385. F4 –F20, fraction 4 – 20; crude, crude methanol extract from rice. TLC sheet was corresponding to figure S3. Solvent system was chloroform: methanol (9:1). X; starting point, * standard antibiotics accidently drop on the agar plate. A, B, C, D and E; compound CA, CB, CC, CD and CE.

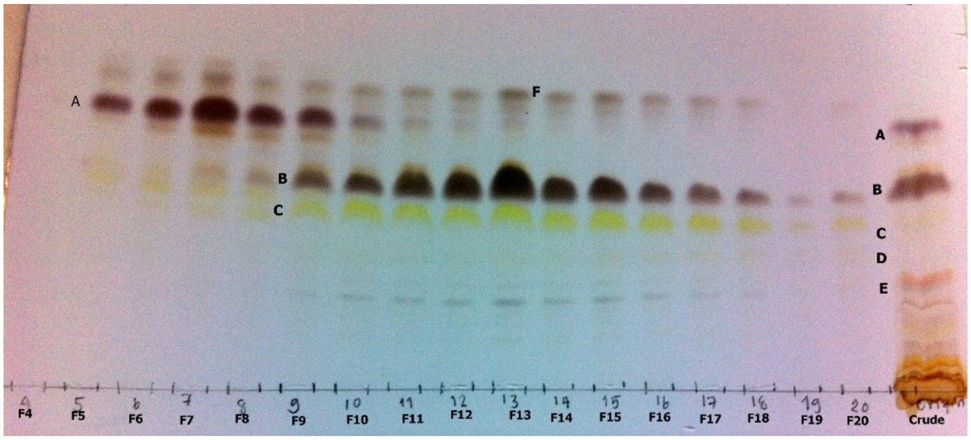

Supplementary figure S3 TLC on silicagel 60 F_{254} of fraction 4-20 from silica gel column of bioactive compounds of strain TML10^{T}; F4 –F20, fraction 4 – 20; crude, crude methanol extract from rice. solvent system was chloroform: methanol (9:1). A, B, C, D, E and F; compound CA, CB, CC, CD CE and CF.

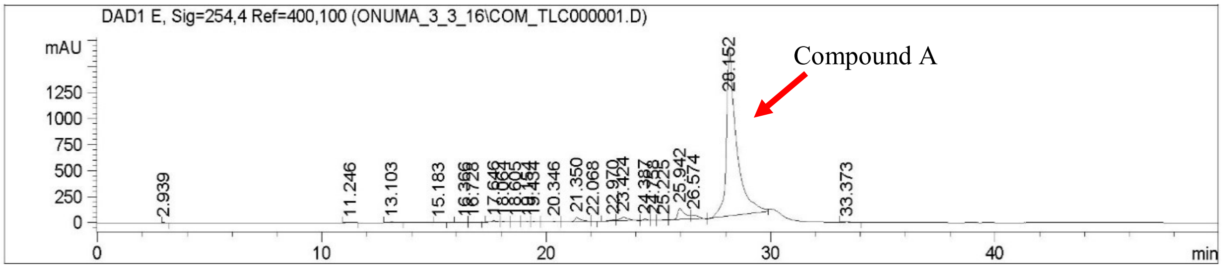

A)

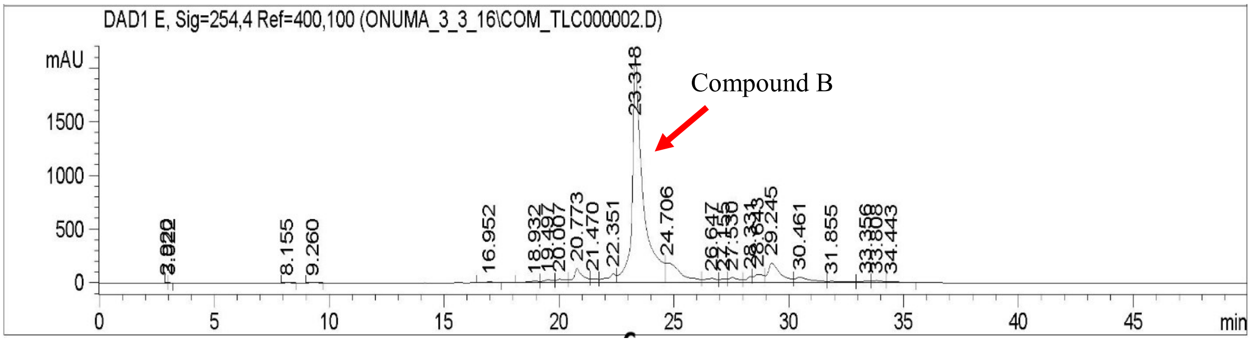

B)

Supplementary figure S4. HPLC analysis of bioactive compounds of strain TML10^{T}. A); compound A (retention time 28.15 min) and B); compound B (retention time 23.31 min) (red arrows) purified by silica gel 60 column and further prepared by preparative TLC.

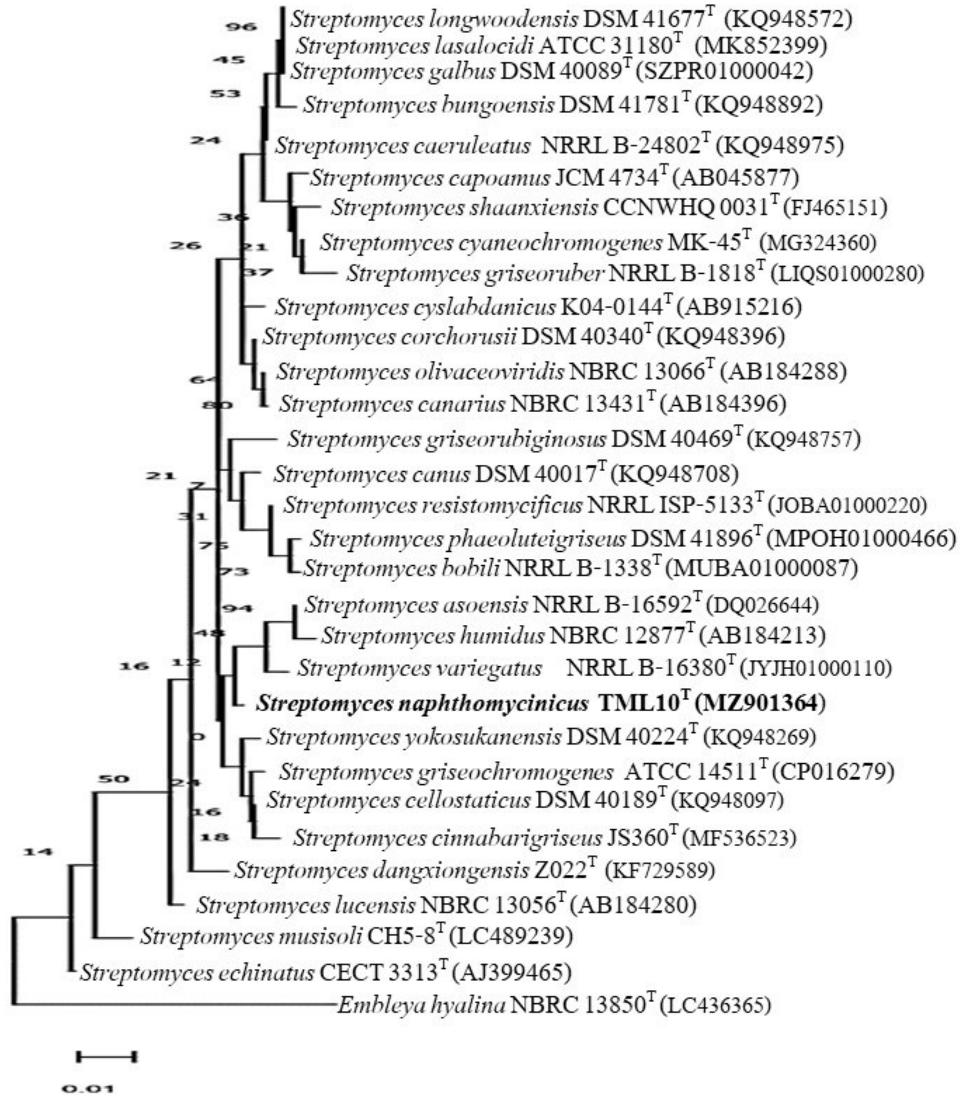

Supplementary figure S5 Maximum-likelihood phylogenetic tree based on 16S rRNA gene sequences (1368 bp) of *Streptomyces naphthomycinicus* TML10^T and their nearest phylogenetic neighbours in genus *Streptomyces* and *Embleya hyalina* NBRC 13850$^{T\,T}$ as the out-group. Bootstrap values based on 1000 replicates are shown at the branch nodes.

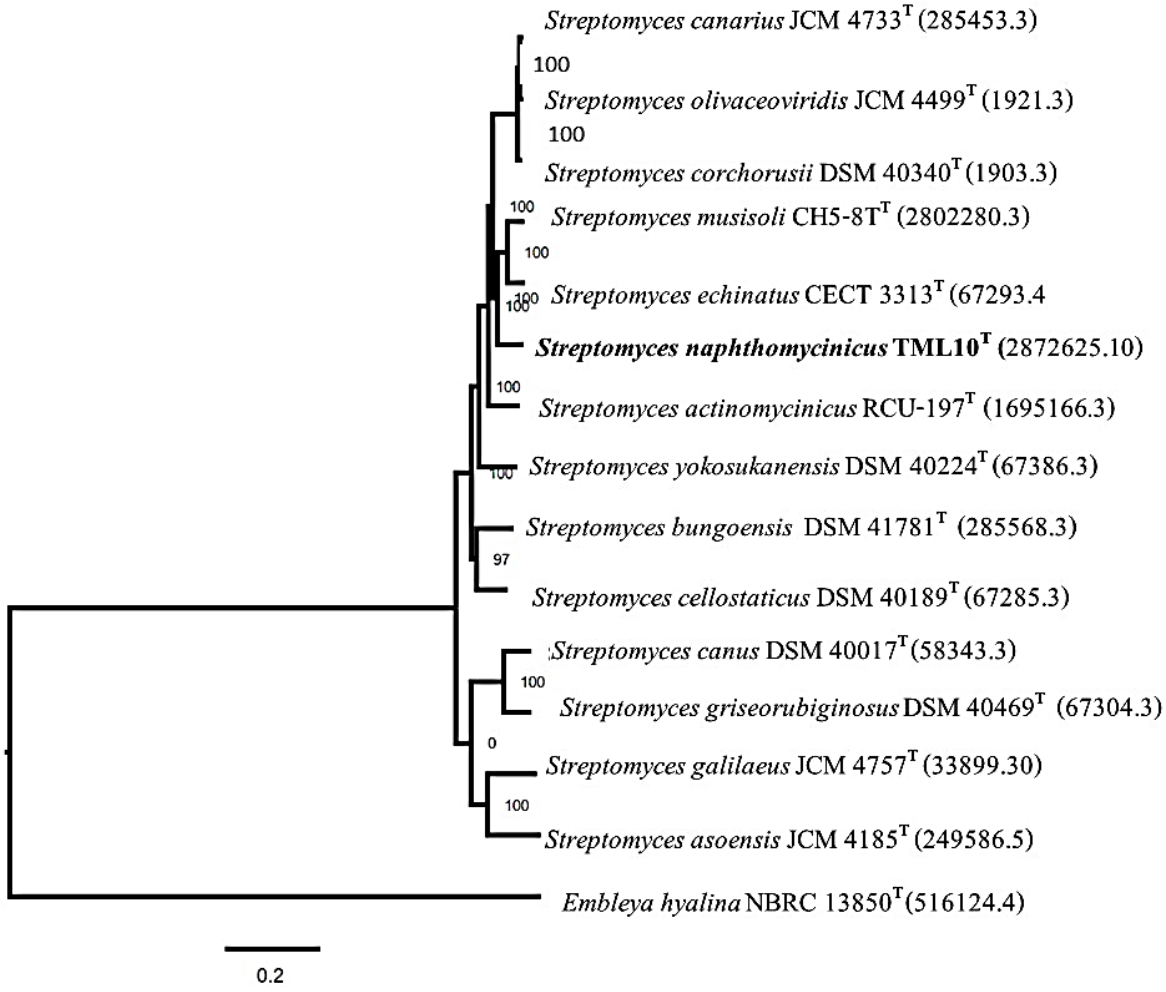

Supplementary figure S6. The maximum likelihood (ML) phylogenomic tree using the codon tree option in the PATRIC webserver of the genomes of strain TML10^{T} and closely related type strains with valid names of *Streptomyces* with *Embleya hyalina* NBRC 13850^{T} as the outgroup.

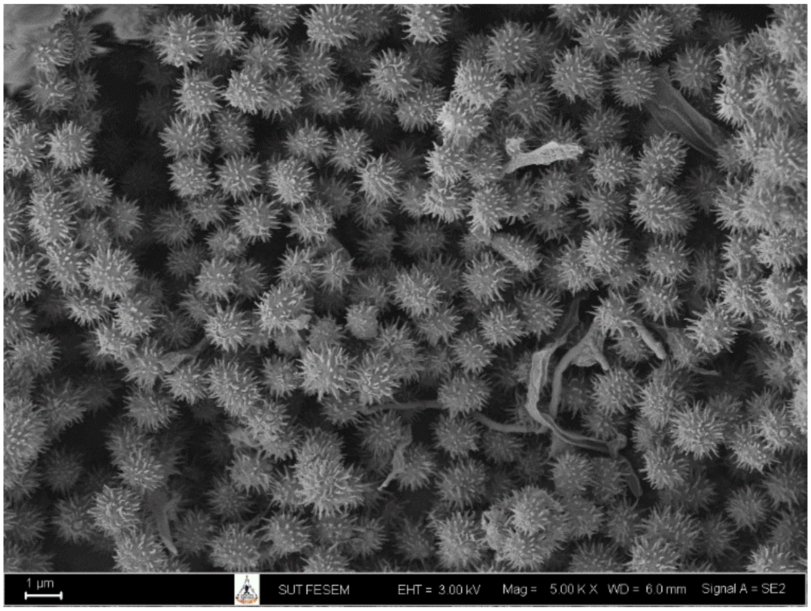

Supplementary Fig. S7 Scanning electron microscope showed spores of strains TML10^T grown on HPDA for 7 days at 27°C showing round rod-shaped spores with spiny surface and spore chains in loop forms. Bar represents 1 µm.

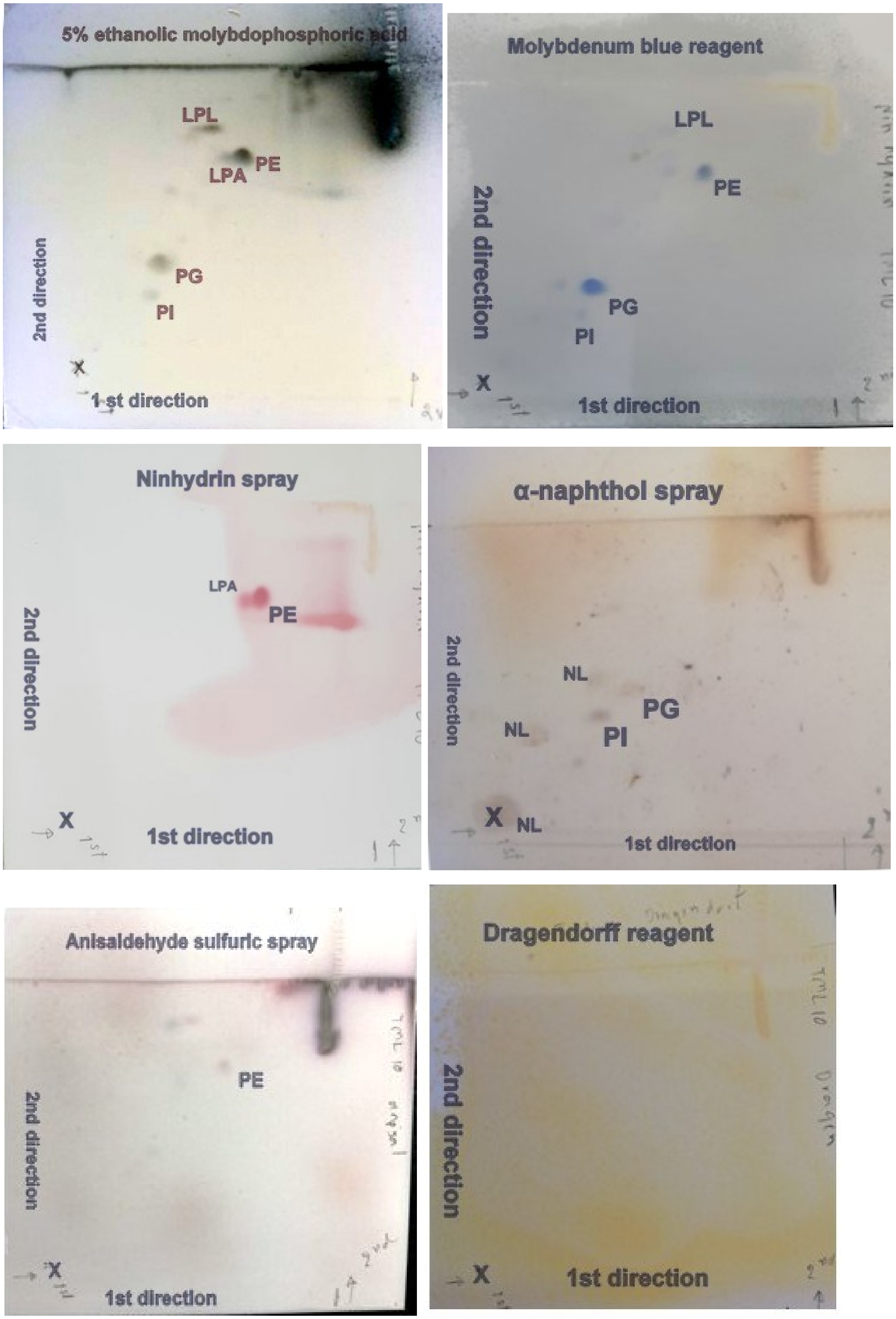
5% ethanolic molybdophosphoric acid
LPL
PE
LPA
PG
PI
2nd direction
X
1 st direction
Molybdenum blue reagent
LPL
PE
2nd direction
PG
PI
X
1st direction
Ninhydrin spray
LPA
PE
2nd direction
X
1st direction
α-naphthol spray
NL
PG
NL
PI
2nd direction
X
NL
1st direction
Anisaldehyde sulfuric spray
PE
2nd direction
X
1st direction
Dragendorff reagent
2nd direction
X
1st direction

Supplementary Fig. S8 Two-dimensional thin-layer chromatography of polar lipids of a) *Streptomyces naphthomycinicus* TML10^T. Chloroform-methanol-water (65:25:4) was used in the first direction, followed by chloroform-acetic acid-methanol-water (40:7.5:6:2) in the second direction. Abbreviations: PE, phosphatidylethanolamide; PG, phosphatidylglycerol; PI, phosphatidylinositol; LPA, unknown lipids with amino group; LPL, unknown lipid with phosphate group; NL, not lipid; X, start point.

Compound A

Naphthomycin A

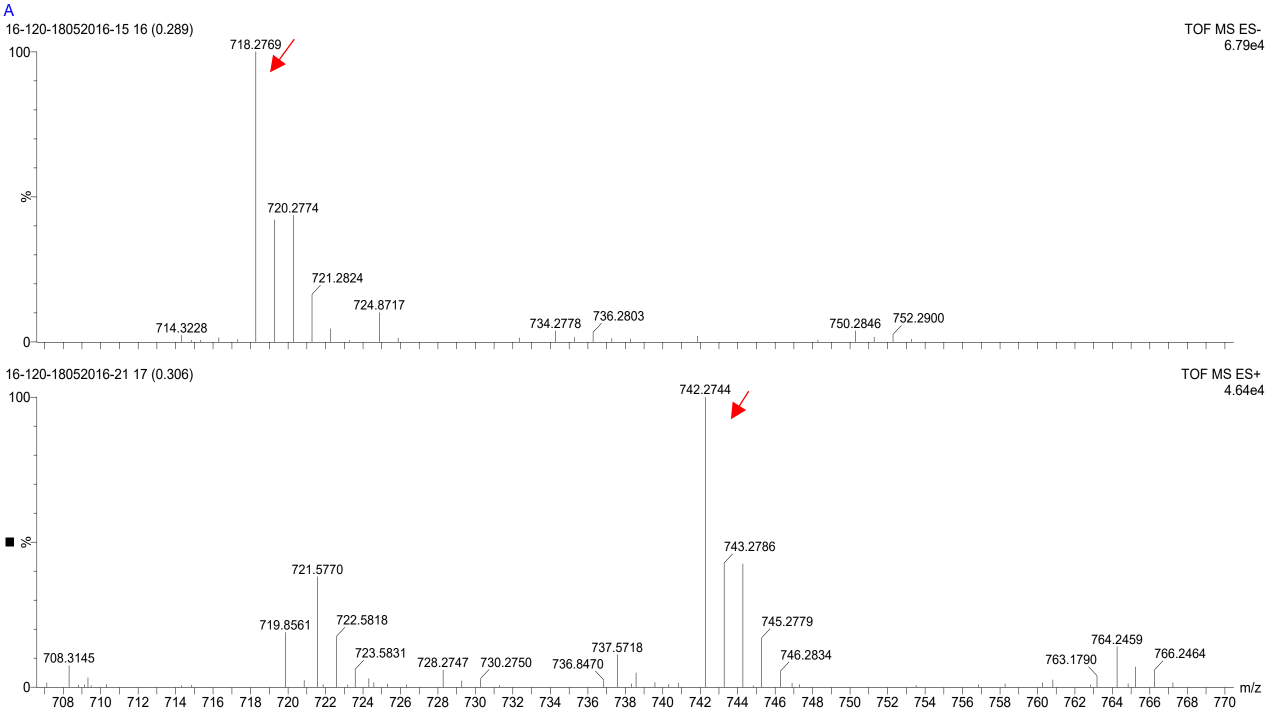

A)

Compoud B CB

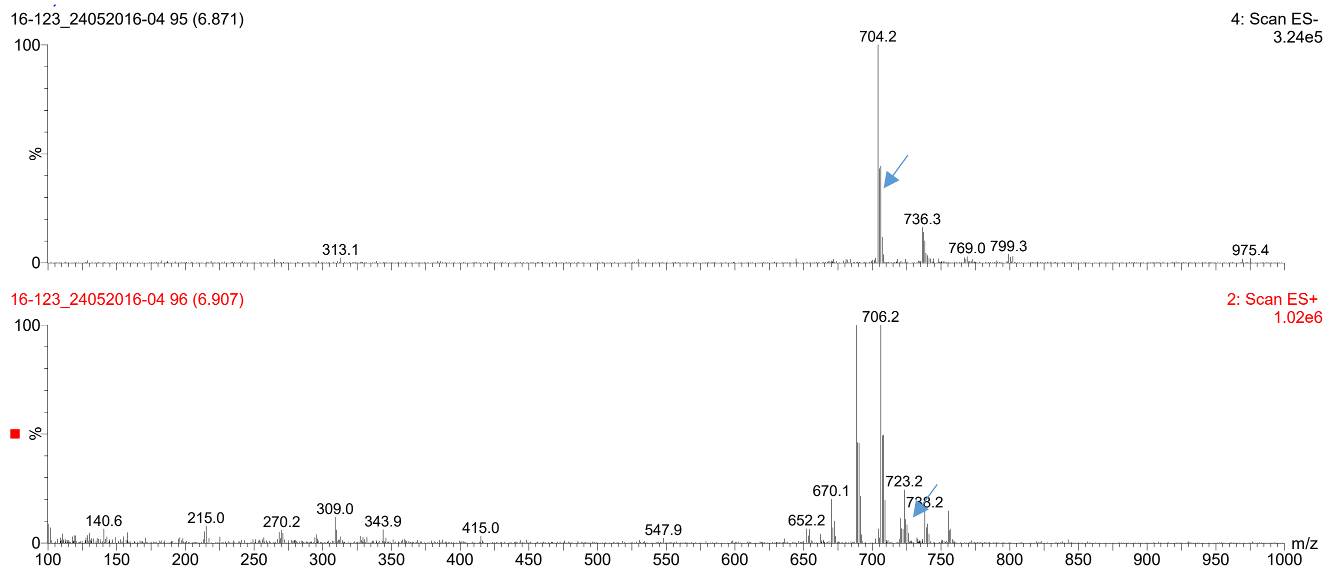

B)

Supplementary figure S9. Reverse phase Liquid Chromatography-Mass Spectrometry (LC-MS) employing UV detection and electrospray mass spectrometry (ESI). A) Molecular weight of compound A (CA) was (M+H) = 718; (M+Na) = 742 (red arrows), and B) compound B (CB) was (M+H) = 706; (M+Na) = 728 (blue arrows).

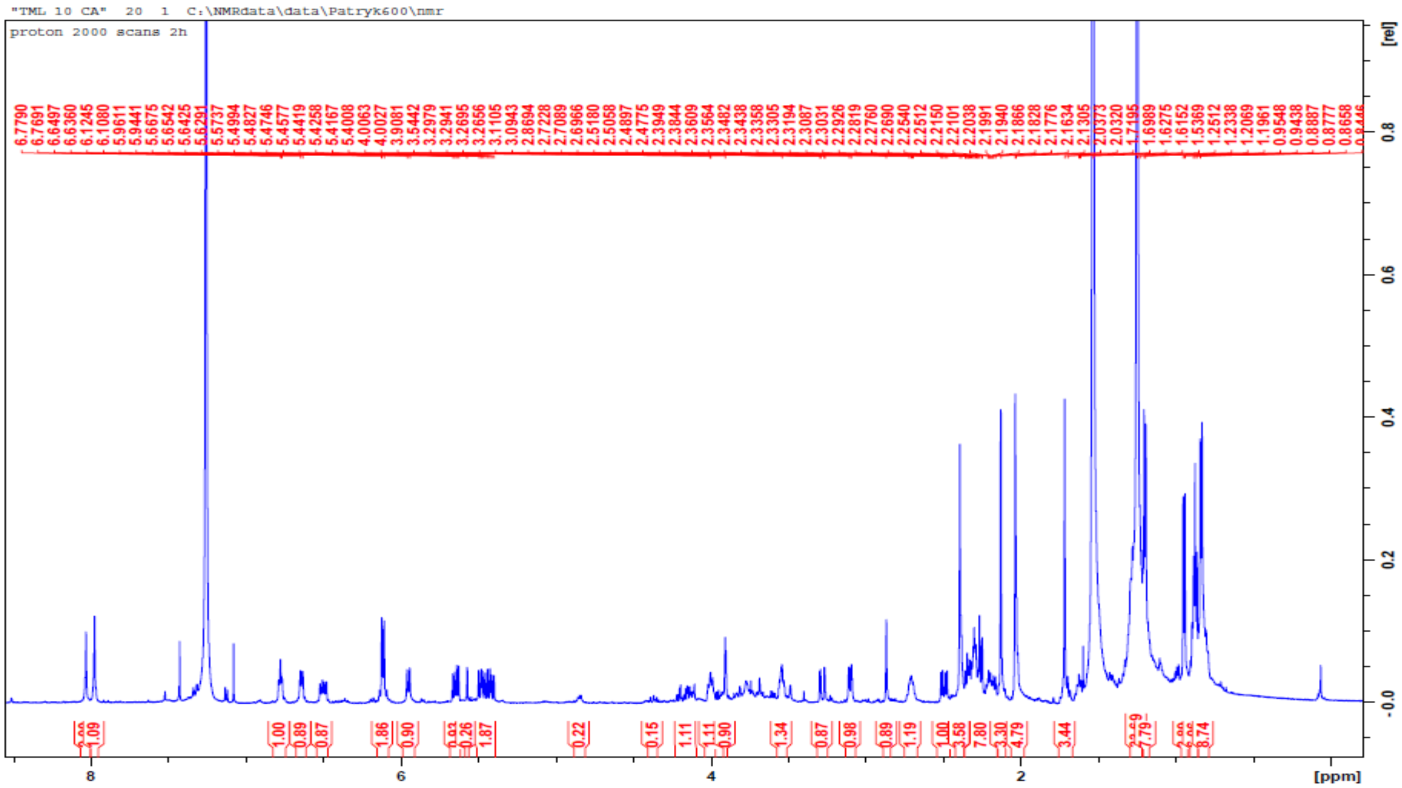

Supplementary Figure S10 A) ^{1}H NMR spectrum of compound A in $CDCl_3$

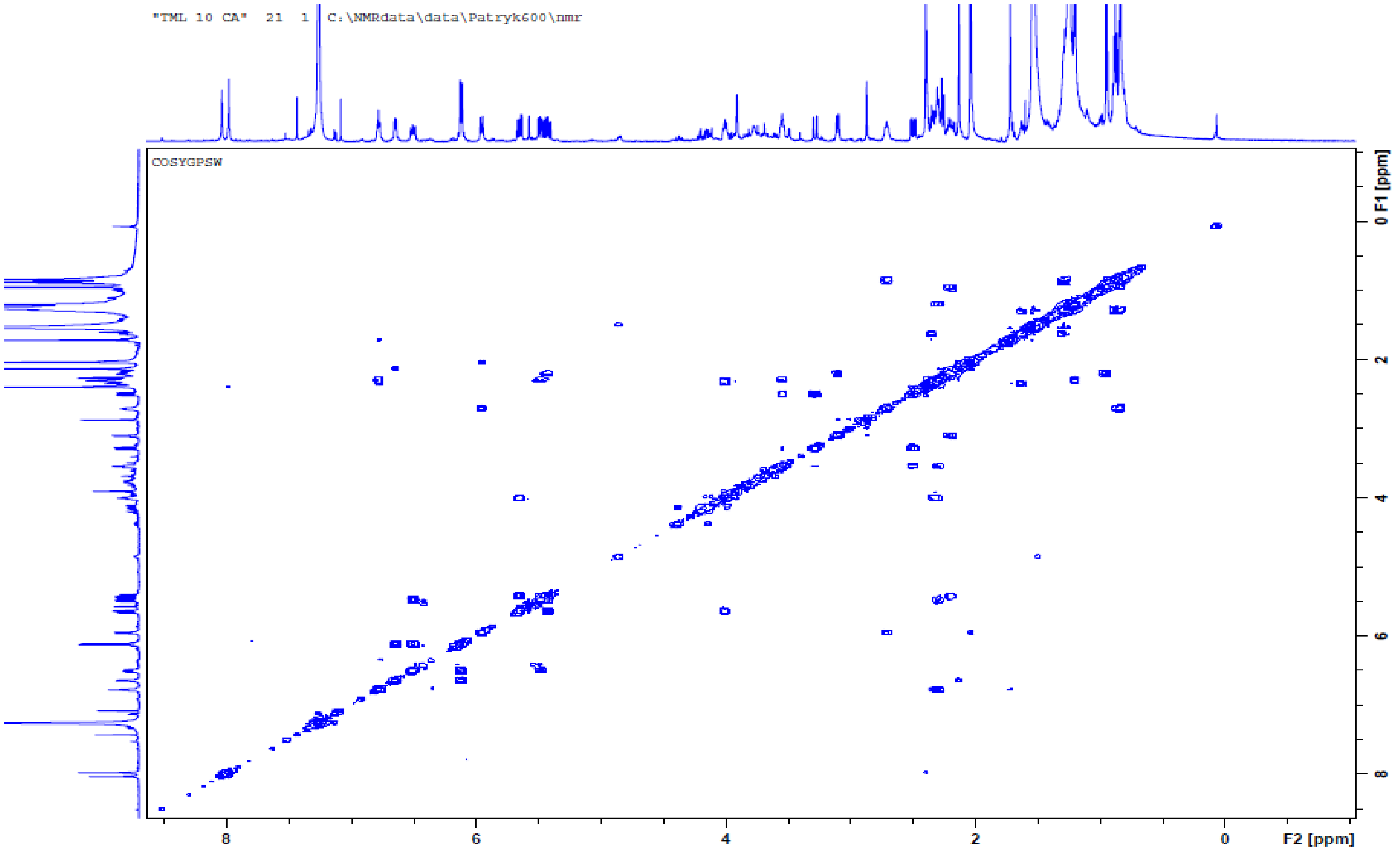

Supplementary Figure S10 B) ^{1}H COSY NMR spectrum of compound A in $CDCl_3$

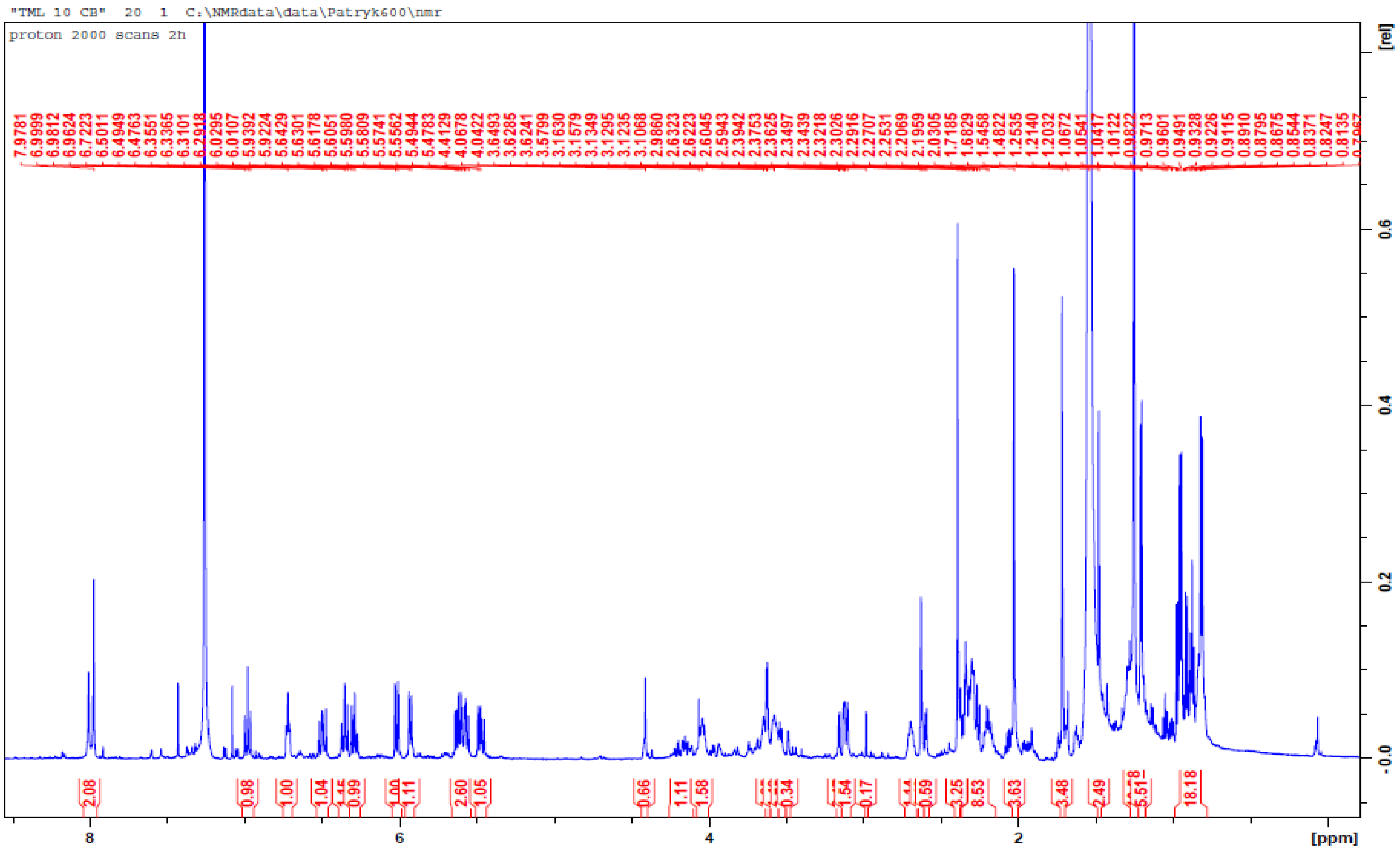

Supplementary Figure S10 C) ^{1}H NMR spectrum of compound B in $CDCl_3$

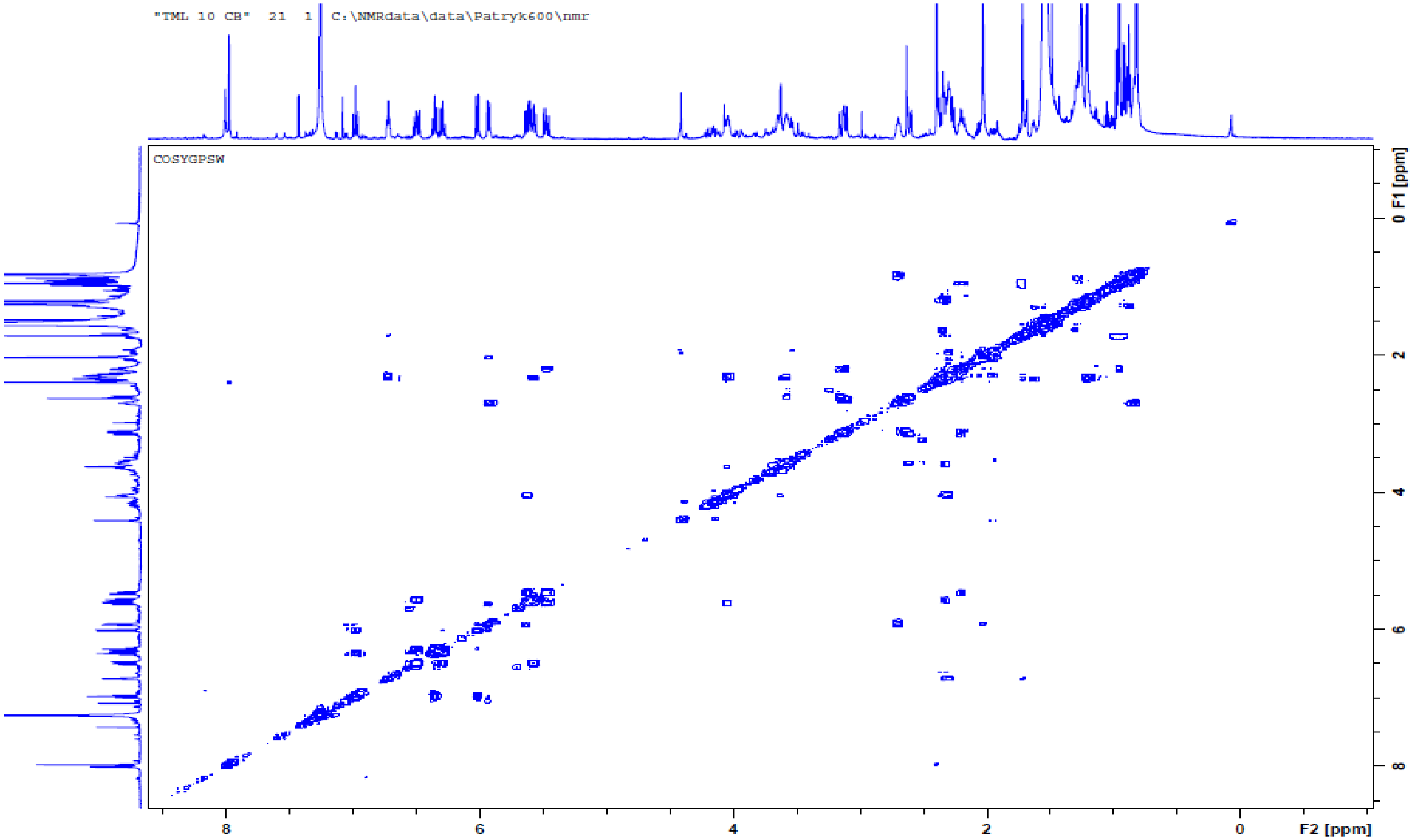

Supplementary Figure S10 D) ^{1}H COSY NMR spectrum of compound B in $CDCl_3$
