# Supplementary material for: Description of Streptomyces naphthomycinicus sp. nov., an endophytic actinobacterium producing naphthomycin A and its genome insight for discovering bioactive compounds
Source: Front Microbiol. 2024 Apr 17;15:1353511. doi: 10.3389/fmicb.2024.1353511 (PMC11061393; doi:10.3389/fmicb.2024.1353511)
Supplement: Supplementary file 2 [file Data_Sheet_2.PDF]

Supplementary Tables

Description of *Streptomyces naphthomycinicus* sp. nov., an endophytic actinobacterium producing naphthomycin A and its genome insight for discovering bioactive compounds

Onuma Kaewkla[1,2*], Mike Perkins[3], Arinthip Thamchaipenet[4], Weerachai Saijuntha[1,5], Sudarat Sukpanoa[6], Chanwit Suriyachadkun[7], Nitcha Chamroensaksi[8], Theeraphan Chumroenphat[9], Christopher Milton Mathew Franco[2]

[1] Center of Excellence in Biodiversity Research, Mahasarakham University, Maha Sarakham 44150, Thailand

[2]Department of Medical Biotechnology, College of Medicine and Public Health, Flinders University, Adelaide, Australia

[3]Deparment of Chemistry, College of Science and Engineering, Flinders University, Adelaide, Australia

[4]Department of Genetics, Kasetsart University, Chatuchak, Bangkok 10900, Thailand

[5] Faculty of Medicine, Mahasarakham University, Maha Sarakham 44000, Thailand

[6]Department of Biology, Faculty of Science, Mahasarakham University, Maha Sarakham 44150, Thailand

[7]Thailand Bioresource Research Center (TBRC), National Center for Genetic Engineering and Biotechnology, National Science and Technology Development Agency, Klong Luang, Pathumthani 12120, Thailand

[8]National Biobank of Thailand (NBT), National Center for Genetic Engineering and Biotechnology, National Science and Technology Development Agency, Klong Luang, Pathumthani 12120, Thailand

[9]Aesthetic Sciences and Health Program, Faculty of Thai Traditional and Alternative Medicine, Ubon Ratchathani Rajabhat University, Ubon Ratchathani 34000, Thailand

* Corresponding author:
Onuma Kaewkla
Email address: Onuma.k@msu.ac.th

Table S1 Cultural properties of *Streptomyces naphthomycinicus* TML10^T following growth on standard media for 7 days at 27 ºC.

Medium/ Strain	Growth (Good /poor)	Aerial mycelium (color)	Substrate mycelium (color)
**Strain TML10^T**			
ISP 2	Good	Grayish green	Brown
ISP 3	Good	Greenish gray	Greenish yellow
ISP 4	Good	Grayish brown	Dark yellow
ISP 5	Good	Yellowish white	Yellow
ISP 7	Good	Grayish green	Dark brown with melanin pigment
Bennett's agar	Good	Grayish green	Olive greeen
1/2 Potato Dextrose agar	Good	Brownish gray	Dark brown
Nutrient agar	Moderate	white	Light yellow

Table S2. Whole-cell fatty acid composition (%) of *Streptomyces naphthomycinicus* TML10^T
Only fatty acids detected at more than 0.1 % of the total are presented. -, not detected. Bold presents fatty acid more than 10%.

**Fatty acids**	Percent
anteiso-$C_{13:0}$	0.2
iso-$C_{14:0}$	1.9
$C_{14:0}$	0.6
iso- $C_{15:1}$ G	0.3
iso-$C_{15:0}$	8.5
anteiso-$C_{15:0}$	**23.8**
$C_{15:1}$ w8c	0.3
$C_{15:1}$ w6c	1.4
iso-$C_{16:1}$ H	1.1
iso-$C_{16:0}$	**15.3**
$C_{16:0}$	7.8
anteiso-$C_{17:1}$ w9c	2.6
iso-$C_{17:1}$ w10c	-
iso-$C_{17:0}$	5.7
anteiso-$C_{17:0}$	**20.1**
$C_{17:1}$ w8c	0.4
$C_{17:0}$ cyclo	0.4
$C_{17:0}$	0.7
$C_{16:1}$ 2OH	0.4
iso-$C_{18:1}$ H	0.4
iso-$C_{18:0}$	0.4
$C_{18:1}$ w9c	1.2
$C_{18:0}$	1.2
iso-$C_{20:0}$	1.9
$C_{16:1}$ w7c	1.8
$C_{18:1}$ w7c	0.3
$C_{16:0\ 10\text{-methyl}}$	1.6

Table S3. Genes encoding proteins relating to bioactive compound production, drought and salt tolerance, stress protein, polysaccharide degradation, lipase, protease, chitin and xylan degradation, phosphatase, xenobiotic degradation and detoxification of *Streptomyces naphthomycinicus* TML10^T.

product	Span (nt)	Score	Percent similarity	Closest Match
**Bioactive compounds production**				
Ketosynthase chain-length factor	3623-4837	1,947	93.3%	*Streptomyces* sp. FBKL.4005
Actinorhodin polyketide synthase	4883-5152	403	93.3%	*Streptomyces* sp. FBKL.4005
Polyketide beta-ketoacyl synthase	32928-34172	1,997	94.0%	*Streptomyces* sp. NRRL B-3648
Chondramide synthase (cmdD)	362719-363327	671	70.4%	*Streptomyces netropsis*
Linear gramicidin synthase subunit D	367011-372173	7,139	81.1%	*Streptomyces netropsis*
Linear gramicidin synthase subunit D	381028-383061	2,324	72.1%	*Streptomyces netropsis*
Tyrocidine synthase III	384595-386391	2,438	81.1%	*Streptomyces netropsis*
Non-ribosomal peptide synthetase	504183-515009	17,339	93.5%	*Streptomyces flaveolus*
Non-ribosomal peptide synthetase	410723-419599	14,109	92.9%	*Streptomyces corchorusii*
Putative non-ribosomal peptide synthetase	9922-13053	2,114	45.6%	*Nocardiopsis* sp. JB363
Dimodular nonribosomal peptide synthase	119-5851	5,706	61.4%	*Streptomyces* sp. F-1
NRPS protein	21403-22977	2,494	91.0%	*Streptomyces hygroscopicus* subsp. *jinggangensis* (strain 5008)
Non-ribosomal peptide synthase protein (TIGR01720 family)	215-1807	1,525	60.4%	*Streptomyces* sp. BK340
Non-ribosomal peptide synthase	149-1684	2,509	94.3%	*Streptomyces hygroscopicus* subsp. *jinggangensis* (strain 5008)
**Drough/ salt tolerance**				
Ectoine dioxygenase	345881-346792	1,482	89.1%	*Streptomyces corchorusii*
L-ectoine synthase	87-491	644	88.1%	*Streptomyces turgidiscabies*
Ectoine dioxygenase	1056-1946	1,370	87.8%	*Streptomyces turgidiscabies* Car8
Ectoine dioxygenase	66828-67721	1,485	93.9%	*Streptomyces hygroscopicus* subsp. *jinggangensis* (strain 5008)
L-ectoine synthase	67727-68131	713	97.8%	*Streptomyces pluripotens*
Glycine/betaine ABC transporter ATPase	62300-63430	1,797	94.9%	*Streptomyces* sp. NRRL B-3648
Glycine betaine/L-proline transporter ProP	82021-83394	2,200	94.3%	*Streptomyces* sp. SID161
Sodium:solute symporter	175612-177072	2,368	96.5%	*Streptomyces bungoensis*
Proline dehydrogenase	95905-96831	1,513	97.4%	*Streptomyces hygroscopicus* subsp. *jinggangensis* (strain 5008)
Osmoprotectant transport system substrate-binding protein	16618-17559	1,426	88.5%	*Streptomyces sp.* BK340

Osmoprotectant transport system permease protein	63427-64080	1,068	98.6%	*Streptomyces* sp. BK387
**Stress**				
Stress-inducible protein	158679-159545	1,346	93.1%	*Streptomyces hygroscopicus* subsp. *jinggangensis* (strain 5008)
Stress-inducible protein	150494-151399	1,207	78.7%	*Streptomyces corchorusii*
Chemical-damaging agent resistance protein C	118276-118854	987	99.0%	*Streptomyces* sp. NRRL B-3648
Tellurium resistance protein	118950-119525	956	97.4%	*Streptomyces hygroscopicus* subsp. *jinggangensis* (strain 5008)
TerD family protein	433208-433786	954	95.8%	*Streptomyces* sp. SID486
Universal stress protein	16429-17304	1,220	81.0%	*Streptomyces monashensis*
UspA domain-containing protein	33610-34482	1,049	72.9%	*Actinobacteria bacterium* OK006
Universal stress protein	37974-38852	1,085	76.0%	*Streptomyces* sp. RB17
Universal stress protein	39019-39462	661	88.4%	*Streptomyces incarnatus*
Universal stress protein	146532-147410	1,286	83.2%	*Streptomyces* sp. RLB1-33
Universal stress protein	151473-152381	1,247	80.8%	*Streptomyces antibioticus*
Ser/Thr protein kinase RdoA (MazF antagonist)	221017-222057	1,724	95.0%	*Streptomyces* sp. BK340
Chemical-damaging agent resistance protein C	188112-188687	976	99.0%	*Streptomyces bungoensis*
**Chaperon and alkaline, heat and cold shock protein**				
Chaperone protein (DnaJ)	317336-318472	1,984	98.9%	*Streptomyces roseochromogenus* subsp. *oscitans* DS 12.976
Heme chaperone (HemW)	321893-323125	2,050	93.4%	*Streptomyces* sp. NRRL B-3648
10 kDa chaperonin	305080-305439	595	96.6%	*Streptomyces roseochromogenus* subsp. *oscitas* DS 12.97
10 kDa chaperonin	91564-91872	507	100.0%	*Streptomyces luteoverticilla*
60 kDa chaperonin	169413-171035	2,627	99.8%	*Streptomyces* sp. SID486
Chaperonin GroEL	91992-93620	2,613	97.0%	*Streptomyces echinatus*
HSP90 family protein	166854-168692	2,953	93.3%	*Streptomyces* sp. SID161
Chaperone protein (HtpG)	60533-62452	3,115	94.1%	*Streptomyces* corchorusii
Chaperone protein DnaK	26309-28153	3,040	98.7%	*Streptomyces* sp. jing01
Chaperone protein DnaK	17977-19881	3,125	98.4%	*Streptomyces corchorusii*
Molecular chaperone (DnaJ)	28837-30009	1,986	97.2%	*Streptomyces echinatus*
Chaperone protein (ClpB)	37462-40059	4,160	96.1%	*Streptomyces misionensis*
Chaperone protein ClpB	21783-24422	4,318	98.9%	*Streptomyces hygroscopicus* subsp. *jinggangensis* (strain 5008)
Alkaline shock protein 23	53489-53959	717	92.9%	*Streptomyces* sp. RB17
Cold-shock protein	341784-341987	381	100.0%	*Streptomyces regalis*
Cold shock protein	171370-171573	380	100.0%	*Streptomyces coelicolor* (strain ATCC BAA-471 / A3(2) / M145)
Cold-shock protein	38610-38816	377	98.5%	*Streptomyces* sp. CB01883
Cold-shock protein	2292-2495	387	100.0%	*Streptomyces* sp. Act143
Cold-shock protein	79960-80163	383	100.0%	*Streptomyces* sp. CB01373
heat shock protein HspR	30015-30464	738	99.3%	*Streptomyces puniciscabiei*
HSP20 family protein	57978-58409	715	98.6%	*Streptomyces* sp. cf386
**Protease**				

Extracellular small neutral protease	40303-40992	1,026	89.3%	*Streptomyces* sp. NRRL B-3648
Neutral metalloproteinase	288837-289907	1,765	94.1%	*Streptomyces* sp. Ru71
Peptidase C69	24277-25800	2,567	96.8%	*Streptomyces antibioticus*
Serine protease	45742-46926	1,797	90.9%	*Streptomyces hygroscopicus* subsp. *jinggangensis* strain 5008
Zn-dependent protease	51762-52562	1,325	94.3%	*Streptomyces echinatus*
Putative zinc protease	6336-7715	2,257	95.2%	*Streptomyces* sp. F-1
**Lipase**				
Lipase	201898-202770	1,335	87.2%	*Streptomyces antibioticus*
**Amylase**				
Alpha-amylase	6684-8384	2,795	90.3%	*Streptomyces* sp. DSM 40868
Alpha-amylase	7188-8564	2,324	92.4%	*Streptomyces* echinatus
**Chitin degradation**				
Chitin-binding protein	2910-3989	1,752	87.5%	*Streptomyces diastatochromogenes*
Non-reducing end alpha-L-arabinofuranosidase	85423-86847	2,448	94.5%	*Streptomyces* sp. Ag109_O5-1
Chitosanase	87417-88256	1,341	91.8%	*Streptomyces* sp. BK340
Chinitase A	55925-57643	2,767	91.6%	*Streptomyces collinus* (strain DSM 40733 / Tue 365)
Chitinase C	25429-27249	3,141	95.2%	*Streptomyces hygroscopicus* subsp. *jinggangensis* (strain 5008)
Xalan and arabinofuranosidase				
Xylose isomerase	72064-73230	1,989	97.7%	*Streptomyces monashensis*
Beta-xylanase	247961-248242	311	84.9%	*Streptomyces scabichelini*
Non-reducing end alpha-L-arabinofuranosidase	85423-86847	2,448	94.5%	*Streptomyces* sp. Ag109_O5-1
**Phosphatase**				
Alkaline phosphatase	366675-368222	2,673	96.3%	*Streptomyces* sp. NRRL B-3648
Phosphatase	213565-214215	1,030	94.9%	*Streptomyces* sp. PBH53
**Biodegradation of xenotic compounds**				
Alkanesulfonate monooxygenase SsuD	132832-134034	2,002	96.8%	*Streptomyces echinatus*
Alkanesulfonate monooxygenase SsuD	39500-40528	1,708	96.5%	*Streptomyces* sp. BK387
Nitroreductase	5912-6583	1,070	88.7%	*Streptomyces* sp. GS7
Nitronate monooxygenase	316047-317135	1,683	88.6%	*Streptomyces* sp. Z022
Epoxide hydrolase	126349-127188	1,259	85.3%	*Streptomyces* sp. MMG1121
Epoxide hydrolase	405779-406768	1,699	96.4%	*Streptomyces agglomeratus*
2-haloacid dehalogenase	84026-84721	965	82.5%	*Streptomyces echinatus*
**Hopene production**				
Squalene-hopene/tetraprenyl-beta-curcumene cyclase	51350-53380	3,463	93.9%	*Streptomyces echinatus*
Squalene-associated FAD-dependent desaturase	54624-56051	2,288	94.1%	*Streptomyces* sp. BK387
**Carotenoid production**				
Phytoene synthase	90466-91494	1,529	89.8%	*Streptomyces* sp. BK447
Phytoene synthase	56176-57138	1,536	94.7%	*Streptomyces yokosukanensis*

Phytoene desaturase	91491-93086	2,491	89.6%	*Streptomyces* sp. BK308
Isorenieratene synthase	96778-98370	2,410	90.8%	*Streptomyces* sp. BK308
**Arginine deiminase**				
Arginine deiminase	1327090-1328313	1480	97.3	*Streptomyces echinatus*
Ornithine carbamoyltransferase	1326005-1327027	1645	95	*Streptomyces* sp. SID161
Ornithine decarboxylase	63139-64611	2205	85.8	*Streptomyces caeruleatus*
Arginine repressor	399923-400468	895	99.4	*Streptomyces* sp. NRRL B-3648

Table S4. Protein alignment of arginine deiminase. Multiple sequence alignment of *Streptomyces* sp. strains TML10^{T} and 13 strains of closely related strains of *Streptomyces* sp and *Bacillus licheniformis*. Red color amino acids (aa) are conserved and are known to be essential for full enzymatic activity of the protein (Das et al., 2004). Dots mark the conservative substitutions of strain TML10^{T}. Green color shows the mutation points of strain TML10^{T}. $^{\#}$ indicates percent similarity of protein sequencing between strain TML10^{T} and others in this study. *; the position of amino acid (1 -421).

Strains	PS (%)#	1*									10*										20*										30*										40*										50*
Streptomyces TML10	100	M	G	F	H	V	D	S	E	A	G	R	L	R	R	V	I	L	H	R	P	D	L	E	L	K	R	L	T	P	S	N	K	D	A	L	L	F	D	D	V	L	W	V	R	R	A	R	A	E	H
Streptomyces echinatus	97.3	.	.	.	.	.	.	.	.	.	.	.	.	.	.	.	.	.	.	.	.	.	.	.	.	.	.	.	.	.	.	.	.	.	.	.	.	.	.	.	.	.	.	.	.	.	.	.	.	.	.
Streptomyces sp. PBH53	96.1	.	.	.	.	.	.	.	.	.	.	.	.	.	.	.	.	.	.	.	.	.	.	.	.	.	.	.	.	.	.	.	.	.	.	.	.	.	.	.	.	.	.	.	.	.	.	.	.	.	.
Streptomyces sp. NRRL B-3648	96.1	.	.	.	.	.	.	.	.	.	.	.	.	.	.	.	.	.	.	.	.	.	.	.	.	.	.	.	.	.	.	.	.	.	.	.	.	.	.	.	.	.	.	.	.	.	.	.	.	.	.
Streptomyces hygroscopicus subsp. jinggangensis (strain 5008)	96.1	.	.	.	.	.	.	.	.	.	.	.	.	.	.	.	.	.	.	.	.	.	.	.	.	.	.	.	.	.	.	.	.	.	.	.	.	.	.	.	.	.	.	.	.	.	.	.	.	.	.
griseochromogenes	95.1	.	A	Y	.	.	.	.	.	V	.	.	.	.	.	.	.	.	.	.	.	.	.	.	.	.	.	.	.	.	.	.	.	.	.	.	.	.	.	.	.	.	.	.	.	.	.	.	.	.	.
Streptomyces sp. SID4985	94.8	.	A	.	.	.	.	.	.	.	.	.	.	.	.	.	.	.	.	.	.	.	.	.	.	.	.	.	.	.	.	.	.	.	.	.	.	.	.	.	.	.	.	.	.	.	.	.	.	.	.
Streptomyces sp. CB01883	95.1	.	A	.	.	.	.	.	.	.	.	.	.	.	.	.	.	.	.	.	.	.	.	.	.	.	.	.	.	.	.	.	.	.	.	.	.	.	.	.	.	.	.	.	.	.	.	.	.	.	.
Streptomyces dangxiongensis	94.8	.	.	.	.	.	.	.	.	.	.	.	.	.	.	.	.	.	.	.	.	.	.	.	.	.	.	.	.	.	.	.	.	.	.	.	.	.	.	.	.	.	.	.	.	.	.	.	.	.	.
Streptomyces actinomycinicus	94.8	.	.	.	.	.	.	.	.	.	.	.	.	.	.	.	.	.	.	.	.	.	.	.	.	.	.	.	.	.	.	.	.	.	.	.	.	.	.	.	.	.	.	.	.	.	.	.	.	.	.
Streptomyces cellostaticus	94.6	.	A	Y	.	.	.	.	.	V	.	.	.	.	Q	.	.	.	.	.	.	.	.	.	.	.	.	.	.	.	.	.	.	.	.	.	.	.	.	.	.	.	.	.	.	.	.	.	.	.	.
Streptomyces lucensis JCM	94.6	.	.	.	.	.	.	.	.	.	.	.	.	.	.	.	.	.	.	.	.	.	.	.	.	.	.	.	.	.	.	.	.	.	.	.	.	.	.	.	.	.	.	.	.	.	.	.	.	.	.
Streptomyces seoulensis	93.9	.	A	.	.	.	.	.	.	.	.	.	.	.	.	.	.	.	.	.	.	.	.	.	.	.	.	.	.	.	.	.	.	.	.	.	.	.	.	.	.	.	.	.	.	.	.	.	.	.	.
Bacillus licheniformis	40	T	P	I	.	.	Y	.	.	I	.	P	.	K	T	.	M	.	K	.	.	G	R	.	.	E	N	.	.	.	E	Y	L	E	R	.	.	.	.	.	I	P	F	L	P	A	V	Q	K	.	.

Strains	PS (%)#	51*									60*										70*										80*										90*										100*
Streptomyces TML10	100	D	G	F	A	D	V	L	R	D	R	G	V	A	V	H	L	F	G	D	L	L	A	E	T	L	A	F	P	E	A	R	T	L	V	L	D	R	V	F	D	E	K	E	Y	G	P	-	L	A	T
Streptomyces echinatus	97.3	.	.	.	.	.	.	.	.	.	.	.	.	V	.	.	.	.	.	.	.	.	.	.	.	.	.	L	.	D	.	.	.	.	.	.	.	.	.	.	.	.	.	.	.	.	.	-	.	.	.
Streptomyces sp. PBH53	96.1	.	.	.	.	.	.	.	.	.	.	.	.	E	.	.	.	.	.	.	.	.	.	.	.	.	.	L	.	.	.	.	A	.	.	.	.	.	.	.	.	.	.	.	.	.	.	-	.	.	.
Streptomyces sp. NRRL B-3648	96.1	.	.	.	.	.	.	.	.	.	.	.	.	E	.	.	.	.	.	.	.	.	.	.	A	.	.	L	.	.	.	.	.	.	.	.	.	.	.	.	.	.	.	.	.	.	.	-	.	.	.
Streptomyces hygroscopicus subsp. jinggangensis (strain 5008)	96.1	.	.	.	.	.	.	.	.	.	.	.	.	E	.	.	.	.	.	.	.	.	.	.	.	.	.	L	.	.	.	.	A	.	.	.	.	.	.	.	.	.	.	.	.	.	.	-	.	.	.
Streptomyces griseochromogenes	95.1	.	.	.	.	.	.	.	.	.	.	.	.	.	.	.	.	.	.	.	.	.	T	.	.	.	.	I	.	.	.	.	.	.	.	.	.	.	.	.	.	.	.	.	.	.	.	-	.	.	.
Streptomyces sp. SID4985	94.8	.	.	.	.	.	.	.	.	.	.	.	.	E	.	.	.	.	.	.	.	.	T	.	.	.	.	V	.	.	.	.	A	.	.	.	.	.	.	.	.	.	.	.	.	.	.	-	.	.	.
Streptomyces sp. CB01883	95.1	.	.	.	.	.	.	.	.	.	.	.	.	T	.	.	.	.	.	E	.	.	T	.	.	.	.	L	.	.	.	.	.	.	.	.	.	.	.	.	.	.	.	.	.	.	.	-	.	.	.
Streptomyces dangxiongensis	94.8	.	.	.	.	.	.	.	.	.	.	.	.	E	.	.	.	.	.	.	.	.	.	.	.	.	.	L	.	.	.	.	A	.	.	.	.	.	.	.	.	.	.	.	.	.	.	-	.	.	.
Streptomyces actinomycinicus	94.8	.	.	.	.	.	.	.	.	.	.	.	.	T	.	.	.	.	.	.	.	.	T	D	.	.	E	I	.	.	.	.	A	.	.	.	.	.	.	.	.	.	.	.	.	.	.	-	.	.	.
Streptomyces cellostaticus	94.6	.	.	.	.	.	.	.	.	.	.	.	.	.	.	.	.	.	.	.	.	.	T	.	.	.	.	I	.	G	.	.	S	.	.	.	.	.	.	.	.	.	.	.	.	.	.	-	.	.	.
Streptomyces lucensis JCM	94.6	.	.	.	.	.	.	.	.	.	.	.	.	T	.	.	.	.	.	E	.	.	T	.	.	.	E	I	.	.	.	.	S	.	.	.	.	.	.	.	.	.	.	.	.	.	.	-	.	.	.
Streptomyces seoulensis	93.9	.	.	.	.	.	.	.	.	.	.	.	.	E	.	.	.	.	.	.	.	.	T	.	.	.	.	V	.	.	.	.	.	.	.	.	.	.	.	.	.	.	.	.	.	.	.	-	.	.	.
Bacillus licheniformis	40	.	Q	.	.	E	T	.	K	Q	Q	.	A	E	.	L	Y	L	E	K	.	T	.	.	A	.	D	D	A	L	V	.	E	Q	F	I	.	E	L	L	T	.	S	K	A	D	I	N	G	.	Y

Strains	PS (%)#	101*									110*										120*										130*										140*										150*
Streptomyces TML10	100	D	H	L	R	A	A	F	D	R	M	T	P	D	E	L	A	E	V	L	V	G	G	M	T	K	R	E	F	L	D	A	H	A	E	P	A	S	V	R	F	H	V	M	E	L	D	D	F	L	L
Streptomyces echinatus	97.3	.	.	.	.	.	.	.	.	.	.	.	.	G	.	.	.	.	A	.	.	.	.	.	.	.	.	.	.	.	.	.	.	.	.	.	.	.	.	.	.	.	.	.	D	.	.	.	.	.	.
Streptomyces sp. PBH53	96.1	G	.	.	.	.	.	.	.	.	.	.	.	G	.	.	.	.	A	.	.	.	.	.	.	.	.	.	.	.	A	G	.	.	.	.	T	.	.	.	.	.	.	.	.	.	.	.	.	.	.
Streptomyces sp. NRRL B-3648	96.1	.	.	.	.	.	.	.	.	G	.	.	.	E	.	.	.	.	A	.	.	.	.	.	.	.	.	.	.	.	A	.	.	.	.	.	T	.	.	.	.	.	.	.	.	.	.	.	.	.	.
Streptomyces hygroscopicus subsp. jinggangensis (strain 5008)	96.1	.	.	.	.	.	.	.	.	G	.	.	.	G	.	.	.	.	A	.	.	.	.	.	.	.	.	.	.	.	A	E	.	.	.	.	.	.	.	.	.	.	.	.	.	.	.	.	.	.	.
Streptomyces griseochromogenes	95.1	.	.	.	.	.	.	.	.	G	.	A	.	G	.	.	.	.	A	.	.	.	.	.	.	.	.	.	Y	.	.	.	.	.	.	.	.	.	.	.	.	.	.	.	D	.	.	.	.	.	.
Streptomyces sp. SID4985	94.8	.	.	.	.	.	.	.	.	.	.	A	.	.	T	.	.	.	A	.	.	.	.	.	.	.	.	.	.	.	.	.	Y	.	.	.	T	.	.	.	.	.	A	.	.	.	.	.	.	.	.
Streptomyces sp. CB01883	95.1	.	.	.	.	.	.	.	.	.	.	.	.	G	.	.	.	.	A	.	.	.	.	.	.	.	.	.	.	.	.	G	.	.	.	.	T	.	.	.	.	.	.	.	D	.	.	.	.	.	.
Streptomyces dangxiongensis	94.8	.	.	.	.	.	.	.	.	G	.	A	.	P	.	.	.	.	A	.	.	.	.	.	.	.	.	.	.	.	.	.	.	.	.	.	T	.	.	.	.	.	.	.	D	.	.	.	.	.	.
Streptomyces actinomycinicus	94.8	.	.	.	.	.	T	.	.	.	.	.	.	A	G	.	.	.	A	.	.	.	.	.	.	.	.	.	.	.	A	.	.	.	.	.	T	.	.	.	.	.	.	.	D	.	.	.	.	.	V
Streptomyces cellostaticus	94.6	.	.	.	.	.	.	.	.	G	.	D	.	G	.	.	.	.	A	.	.	.	.	.	.	.	.	.	.	.	.	.	.	.	.	.	T	.	.	.	.	.	.	.	D	.	.	.	.	.	.
Streptomyces lucensis JCM	94.6	G	.	.	.	.	.	.	.	.	.	.	.	G	.	.	.	.	A	.	.	.	.	.	.	.	.	.	.	.	.	.	Y	.	.	.	.	.	.	.	.	.	.	.	D	.	.	.	.	.	.
Streptomyces seoulensis	93.9	.	.	.	.	T	.	.	.	A	.	A	.	G	.	.	.	.	A	.	.	.	.	.	.	.	.	.	.	.	T	G	.	P	.	.	.	.	.	.	.	.	.	.	.	.	.	.	.	.	.
Bacillus licheniformis	40	.	R	.	K	E	F	L	L	T	F	D	A	.	S	M	V	.	Q	V	M	S	.	I	R	.	N	.	L	E	R	-	-	E	K	K	S	H	L	H	E	L	M	E	D	H	Y	P	.	Y	.

Strains	PS (%)#	151*									160*										170*										180*										190*										200*
Streptomyces TML10	100	P	P	L	P	N	H	L	F	T	R	D	T	S	A	W	I	Y	D	G	V	S	I	N	A	M	R	R	P	A	R	Q	R	E	T	V	H	F	E	A	I	Y	R	H	H	P	L	F	R	D	E
Streptomyces echinatus	97.3	.	.	.	.	.	.	.	.	.	.	.	.	.	.	.	.	.	.	.	.	.	.	.	.	.	.	.	.	.	.	.	.	.	.	.	.	.	.	.	.	.	.	.	.	.	.	.	.	.	.
Streptomyces sp. PBH53	96.1	R	.	.	.	.	.	.	.	.	.	.	.	.	.	.	.	.	.	.	.	.	.	.	.	.	.	W	.	.	.	.	.	.	.	.	.	.	.	.	.	.	.	.	.	.	.	.	.	.	.
Streptomyces sp. NRRL B-3648	96.1	R	.	.	.	.	.	.	.	.	.	.	.	.	.	.	.	.	.	.	.	.	.	.	.	.	.	W	.	.	.	.	.	.	.	.	.	.	.	.	.	.	.	.	.	.	.	.	.	.	.
Streptomyces hygroscopicus subsp. jinggangensis (strain 5008)	96.1	R	.	.	.	.	.	.	.	.	.	.	.	.	.	.	.	.	.	.	.	.	.	.	.	.	.	W	.	.	.	.	.	.	.	.	.	.	.	.	.	.	.	.	.	.	.	.	.	.	.
Streptomyces griseochromogenes	95.1	R	.	.	.	.	.	.	.	.	.	.	.	.	.	.	.	.	.	.	.	.	.	.	.	.	.	W	.	.	.	.	.	.	.	.	.	.	.	.	.	.	.	.	.	.	.	.	.	.	.
Streptomyces sp. SID4985	94.8	G	.	.	.	.	.	.	.	.	.	.	.	.	.	.	.	.	.	.	.	.	.	.	.	.	.	W	.	.	.	.	.	.	.	.	.	.	.	.	.	.	.	.	.	.	.	.	.	.	.
Streptomyces sp. CB01883	95.1	R	.	.	.	.	.	.	.	.	.	.	.	.	.	.	.	.	.	.	.	.	.	.	.	.	.	W	.	.	.	.	.	.	.	.	.	.	.	.	.	.	.	.	.	.	.	.	.	A	.
Streptomyces dangxiongensis	94.8	.	.	.	.	.	.	.	.	.	.	.	.	.	.	.	.	.	.	.	.	.	.	.	.	.	.	.	.	.	.	.	.	.	.	.	.	.	.	.	.	.	.	.	.	.	.	.	H	G	.
Streptomyces actinomycinicus	94.8	.	.	.	.	.	.	.	.	.	.	.	.	.	.	.	.	.	.	.	.	.	.	.	.	.	.	.	.	.	.	.	.	.	.	.	.	.	.	.	.	.	.	.	.	.	.	.	.	E	.
Streptomyces cellostaticus	94.6	R	.	.	.	.	.	.	.	.	.	.	.	.	.	.	.	.	.	.	.	.	.	.	.	.	.	W	.	.	.	.	.	.	.	.	.	.	.	.	.	.	.	.	.	.	.	.	.	.	.
Streptomyces lucensis JCM	94.6	R	.	.	.	.	.	.	.	.	.	.	.	.	.	.	.	.	.	.	.	.	.	.	.	.	.	W	.	.	.	.	.	.	.	.	.	.	.	.	.	.	.	.	.	.	.	.	C	A	.
Streptomyces seoulensis	93.9	G	.	.	.	.	.	.	.	.	.	.	.	.	.	.	.	.	.	.	.	.	.	.	.	.	.	.	.	.	.	.	.	.	.	.	.	.	.	.	.	.	.	.	.	.	.	.	E	K	.
Bacillus licheniformis	40	D	.	M	.	.	L	Y	.	.	.	.	P	A	.	A	.	G	S	.	L	T	.	.	K	.	K	E	.	.	.	R	.	.	S	L	F	M	R	Y	.	I	N	.	.	.	R	.	K	G	H

Strains	PS (%)#	201*									210*										220*										230*										240*										250*
Streptomyces TML10	100	T	-	-	-	-	F	R	V	W	S	R	G	Q	A	D	Y	P	S	T	I	E	G	G	D	V	L	V	I	G	D	G	A	V	L	I	G	M	S	E	R	T	T	P	Q	A	V	E	M	L	A
Streptomyces echinatus	97.3	.	-	-	-	-	.	H	.	.	.	.	.	.	.	.	.	.	.	.	.	.	.	.	.	.	.	.	.	.	S	.	.	.	.	.	.	.	.	.	.	.	.	.	.	.	.	.	.	.	.
Streptomyces sp. PBH53	96.1	.	-	-	-	-	.	.	I	.	.	.	.	.	.	.	.	.	.	.	.	.	.	.	.	.	.	.	.	.	N	.	.	.	.	.	.	.	.	.	.	.	.	.	.	.	.	.	.	.	.
Streptomyces sp. NRRL B-3648	96.1	.	-	-	-	-	.	H	.	.	.	.	.	.	.	.	.	.	.	.	.	.	.	.	.	.	.	.	.	.	N	.	.	.	.	.	.	.	.	.	.	.	.	.	.	.	.	.	.	.	.
Streptomyces hygroscopicus subsp. jinggangensis (strain 5008)	96.1	.	-	-	-	-	.	H	.	.	.	.	.	.	.	.	.	.	.	.	.	.	.	.	.	.	.	.	.	.	N	.	.	.	.	.	.	.	.	.	.	.	.	A	.	.	.	.	.	.	.
Streptomyces griseochromogenes	95.1	.	-	-	-	-	.	.	I	.	.	.	.	.	.	.	.	.	.	.	.	.	.	.	.	.	.	.	.	.	N	.	.	.	.	.	.	.	.	.	.	.	.	.	.	.	.	.	.	.	.
Streptomyces sp. SID4985	94.8	.	-	-	-	-	.	G	I	.	.	.	.	.	.	.	.	.	.	.	.	.	.	.	.	.	.	.	.	.	N	.	.	.	.	.	.	.	.	.	.	.	.	.	.	.	.	.	.	.	.
Streptomyces sp. CB01883	95.1	M	-	-	-	-	.	H	.	.	.	.	.	.	.	.	F	.	.	.	.	.	.	.	.	.	.	.	.	.	N	.	.	.	.	.	.	.	.	.	.	.	.	.	.	.	.	.	.	.	.
Streptomyces dangxiongensis	94.8	.	-	-	-	-	.	H	.	.	.	.	.	.	.	.	.	.	.	.	.	.	.	.	.	.	.	.	L	.	G	.	V	.	.	.	.	.	.	.	.	.	.	.	.	.	.	.	.	.	.
Streptomyces actinomycinicus	94.8	.	-	-	-	-	.	.	.	.	.	.	.	.	.	.	.	.	.	.	.	.	.	.	.	.	.	.	.	.	G	.	.	.	.	.	.	.	.	.	.	.	.	.	.	.	.	.	.	.	.
Streptomyces cellostaticus	94.6	.	-	-	-	-	.	H	.	.	.	.	.	.	.	.	.	.	.	.	.	.	.	.	.	.	.	.	.	.	N	.	.	.	.	.	.	.	.	.	.	.	.	.	.	.	.	.	.	.	.
Streptomyces lucensis JCM	94.6	.	-	-	-	-	.	H	.	.	.	.	.	.	.	.	.	.	.	.	.	.	.	.	.	.	.	.	.	.	N	.	.	.	.	.	.	.	.	.	.	.	.	.	.	.	.	.	.	.	.
Streptomyces seoulensis	93.9	G	-	-	-	-	.	N	I	.	.	.	.	.	.	.	H	.	.	.	.	.	.	.	.	.	.	.	.	.	N	.	.	.	.	.	.	.	.	.	.	.	.	.	.	.	.	.	.	.	.
Bacillus licheniformis	40	E	-	-	-	-	I	P	.	.	L	D	R	-	-	.	F	K	F	N	.	.	.	.	.	E	.	.	L	N	E	E	T	.	A	.	.	V	.	.	.	.	.	A	.	.	I	.	R	.	V

Strains	PS (%)#	251*									260*										270*										280*										290*										300*
Streptomyces TML10	100	H	K	L	F	E	A	G	S	-	A	R	T	I	V	A	L	D	M	P	K	R	R	A	F	M	H	L	D	T	V	M	T	M	V	D	G	D	T	F	T	Q	Y	A	G	L	-	-	-	-	G
Streptomyces echinatus	97.3	.	.	.	.	A	.	.	.	-	.	.	.	.	.	.	.	.	.	.	.	.	.	.	.	.	.	.	.	.	.	.	.	.	.	.	.	.	.	.	.	.	.	.	.	.	-	-	-	-	.
Streptomyces sp. PBH53	96.1	.	.	.	.	A	.	.	.	-	.	.	.	.	.	.	.	.	.	.	.	.	.	.	.	.	.	.	.	.	.	.	.	.	.	.	.	.	.	.	.	.	.	.	.	.	-	-	-	-	.
Streptomyces sp. NRRL B-3648	96.1	.	.	.	.	A	.	.	.	-	.	.	.	.	.	.	.	.	.	.	.	.	.	.	.	.	.	.	.	.	.	.	.	.	.	G	.	.	.	.	.	.	.	.	.	.	-	-	-	-	.
Streptomyces hygroscopicus subsp. jinggangensis (strain 5008)	96.1	.	.	.	.	A	.	.	.	-	.	.	.	.	.	.	.	.	.	.	.	.	.	.	.	.	.	.	.	.	.	.	.	.	.	.	.	.	.	.	.	.	.	.	.	.	-	-	-	-	.
Streptomyces griseochromogenes	95.1	Y	.	.	.	A	.	.	.	-	.	.	.	.	.	.	.	.	.	.	.	S	.	.	.	.	.	.	.	.	.	.	.	.	.	.	.	.	.	.	.	.	.	.	.	.	-	-	-	-	.
Streptomyces sp. SID4985	94.8	.	.	.	.	A	.	.	.	-	.	.	.	.	.	.	.	.	.	.	.	.	.	.	.	.	.	.	.	.	.	.	.	.	.	.	.	.	.	.	.	.	.	.	.	.	-	-	-	-	.
Streptomyces sp. CB01883	95.1	.	.	.	.	A	.	.	.	-	.	.	.	.	.	.	.	.	.	.	.	.	.	.	.	.	.	.	.	.	.	.	.	.	.	.	.	.	.	.	.	.	.	.	.	.	-	-	-	-	.
Streptomyces dangxiongensis	94.8	.	.	.	.	A	.	.	.	-	.	.	.	.	.	.	.	.	L	.	.	.	.	.	.	.	.	.	.	.	.	.	.	.	.	.	.	.	I	.	.	.	.	.	.	.	-	-	-	-	.
Streptomyces actinomycinicus	94.8	.	.	.	.	A	.	.	.	-	.	.	.	.	.	.	.	.	.	.	.	.	.	.	.	.	.	.	.	.	.	.	.	.	.	.	.	.	.	.	.	.	.	.	.	.	-	-	-	-	.
Streptomyces cellostaticus	94.6	.	.	.	.	A	.	.	.	-	.	.	A	.	.	.	.	.	.	.	.	.	.	.	.	.	.	.	.	.	.	.	.	.	.	.	.	.	.	.	.	.	.	.	.	.	-	-	-	-	.
Streptomyces lucensis JCM	94.6	.	.	.	.	A	.	.	.	-	.	.	.	.	.	.	.	.	.	.	.	.	.	.	.	.	.	.	.	.	.	.	.	.	.	.	.	.	.	.	.	.	.	.	.	.	-	-	-	-	.
Streptomyces seoulensis	93.9	.	.	.	.	A	.	.	.	-	.	Q	.	.	.	.	.	.	.	.	.	.	.	.	.	.	.	.	.	.	.	.	.	.	.	.	.	.	.	.	.	.	.	.	.	.	-	-	-	-	.
Bacillus licheniformis	40	R	N	.	.	Q	R	Q	.	R	I	.	R	V	L	.	V	E	I	.	.	S	.	.	.	.	.	.	.	.	.	F	.	.	.	.	R	.	Q	.	.	I	H	P	A	I	Q	G	P	E	.

Strains	PS (%)#	301*									310*										320*										330*										340*										350*
Streptomyces TML10	100	M	L	R	S	Y	S	I	E	P	G	-	-	-	V	G	E	R	E	L	K	V	T	D	H	P	P	E	H	M	H	R	A	I	A	A	A	L	G	L	G	E	I	R	-	V	L	T	A	T	Q
Streptomyces echinatus	97.3	.	.	.	.	.	T	.	.	.	.	-	-	-	.	.	.	.	.	.	.	.	.	.	.	.	.	.	.	.	.	.	.	.	.	.	.	.	.	.	.	.	.	.	-	.	.	.	.	.	.
Streptomyces sp. PBH53	96.1	.	.	.	.	.	T	.	.	.	.	-	-	-	T	.	.	.	.	.	.	.	.	.	.	.	.	.	.	.	.	.	.	.	.	.	.	.	.	.	.	.	.	.	-	.	.	.	.	.	.
Streptomyces sp. NRRL B-3648	96.1	.	.	.	.	.	T	.	.	.	.	-	-	-	.	.	.	.	.	.	.	.	.	.	.	.	.	.	.	.	.	.	.	.	.	.	.	.	.	.	.	.	.	.	-	.	.	.	.	.	.
Streptomyces hygroscopicus subsp. jinggangensis (strain 5008)	96.1	.	.	.	.	.	T	.	.	.	.	-	-	-	.	.	.	.	.	.	.	.	.	.	.	.	.	.	.	.	.	.	.	.	.	.	.	.	.	.	.	.	.	.	-	.	.	.	.	.	.
Streptomyces griseochromogenes	95.1	.	.	.	.	.	T	.	.	.	.	-	-	-	.	.	.	.	.	.	.	.	.	.	.	.	.	.	.	.	.	.	.	.	.	.	.	.	.	.	.	.	.	.	-	.	.	.	.	.	.
Streptomyces sp. SID4985	94.8	.	.	.	.	.	T	.	.	.	.	-	-	-	.	.	D	K	.	.	.	.	.	.	.	.	.	.	.	.	.	.	.	.	.	.	.	.	.	.	.	.	.	.	-	.	.	.	.	.	.
Streptomyces sp. CB01883	95.1	.	.	.	.	.	T	.	.	.	.	-	-	-	.	.	.	.	.	.	.	.	.	.	.	.	.	.	.	.	.	.	.	.	.	.	.	.	.	.	.	.	.	.	-	.	.	.	.	.	.
Streptomyces dangxiongensis	94.8	.	.	.	.	.	T	.	.	.	.	-	-	-	.	.	.	.	.	.	.	.	.	.	.	.	.	.	.	.	.	.	.	.	.	.	.	.	.	.	.	.	.	.	-	.	.	.	.	.	.
Streptomyces actinomycinicus	94.8	.	.	.	.	.	T	.	.	A	.	-	-	-	D	.	.	.	.	.	.	.	.	.	.	.	.	.	.	.	.	.	.	.	.	.	.	.	.	.	.	.	.	.	-	.	.	.	.	.	.
Streptomyces cellostaticus	94.6	.	.	.	.	.	T	.	.	.	.	-	-	-	.	.	.	.	.	.	.	.	.	.	.	.	.	.	.	.	.	.	.	.	.	.	.	.	.	.	.	.	.	.	-	.	.	.	.	.	.
Streptomyces lucensis JCM	94.6	.	.	.	.	.	T	.	.	.	.	-	-	-	.	.	.	.	.	.	.	.	.	.	.	.	.	.	.	.	.	.	.	.	.	.	.	.	.	.	.	.	.	.	-	.	.	.	.	.	.
Streptomyces seoulensis	93.9	.	.	.	.	.	T	.	.	.	.	-	-	-	.	.	.	.	.	.	.	.	.	.	.	.	.	.	.	.	.	.	.	.	.	.	.	.	.	.	.	.	.	.	-	.	.	.	.	.	.
Bacillus licheniformis	40	D	M	.	I	F	V	L	.	R	.	-	-	-	K	T	A	D	.	I	H	T	.	E	E	H	-	-	N	L	P	E	V	L	K	R	T	.	.	.	S	D	V	N	L	I	F	C	G	G	G

Strains	PS (%)#	351*									360*										370*										380*										390*										400*
Streptomyces TML10	100	D	V	H	A	A	E	R	E	Q	W	D	D	G	C	N	V	L	A	V	E	P	G	V	V	V	A	Y	E	R	N	S	T	T	N	T	H	L	R	K	Q	G	I	E	V	I	E	I	P	G	S
Streptomyces echinatus	97.3	.	.	.	.	.	.	.	.	.	.	.	.	.	.	.	.	.	.	.	.	.	.	.	.	.	.	.	.	.	.	.	.	.	.	.	.	.	.	.	.	.	.	.	.	.	.	.	.	.	.
Streptomyces sp. PBH53	96.1	.	.	.	.	.	.	.	.	.	.	.	.	.	.	.	.	.	.	.	.	.	.	.	.	.	.	.	.	.	.	.	.	.	.	.	.	.	.	.	.	.	.	.	.	.	.	.	.	.	.
Streptomyces sp. NRRL B-3648	96.1	.	.	.	.	.	.	.	.	.	.	.	.	.	.	.	.	.	.	.	.	.	.	.	.	.	.	.	.	.	.	.	.	.	.	.	.	.	.	.	.	.	.	.	.	.	.	.	.	.	.
Streptomyces hygroscopicus subsp. jinggangensis (strain 5008)	96.1	.	.	.	.	.	.	.	.	.	.	.	.	.	.	.	.	.	.	.	.	.	.	.	.	.	.	.	.	.	.	.	.	.	.	.	.	.	.	.	.	.	.	.	.	.	.	.	.	.	.
Streptomyces griseochromogenes	95.1	.	.	.	.	.	.	.	.	.	.	.	.	.	.	.	.	.	.	.	.	.	.	.	.	.	.	.	.	.	.	.	.	.	.	.	.	.	.	.	.	.	.	.	.	.	.	.	.	.	.
Streptomyces sp. SID4985	94.8	.	.	.	.	.	.	.	.	.	.	.	.	.	.	.	.	.	.	.	.	.	.	.	.	.	.	.	.	.	.	A	.	.	.	.	.	.	.	.	.	.	.	.	.	.	.	.	.	.	.
Streptomyces sp. CB01883	95.1	.	.	.	.	.	.	.	.	.	.	.	.	.	.	.	.	.	.	.	.	.	.	.	.	.	.	.	.	.	.	.	.	.	.	.	.	.	.	.	.	.	.	.	.	.	.	.	.	.	.
Streptomyces dangxiongensis	94.8	.	.	.	.	.	.	.	.	.	.	.	.	.	.	.	.	.	.	.	.	.	.	.	.	.	.	.	.	.	.	V	.	.	.	.	.	.	.	.	C	.	.	.	.	.	.	.	.	.	.
Streptomyces actinomycinicus	94.8	.	.	.	.	.	.	.	.	.	.	.	.	.	.	.	.	.	.	.	.	.	.	.	.	.	.	.	.	.	.	V	.	.	.	.	.	.	.	.	.	.	.	.	.	.	.	.	.	.	.
Streptomyces cellostaticus	94.6	.	.	.	.	.	.	.	.	.	.	.	.	.	.	.	.	.	.	.	.	.	.	.	.	.	.	.	.	.	.	.	.	.	.	.	.	.	.	.	.	.	.	.	.	.	.	.	.	.	.
Streptomyces lucensis JCM	94.6	.	.	.	.	.	.	.	.	.	.	.	.	.	.	.	.	.	.	.	.	.	.	.	.	.	.	.	.	.	.	A	.	.	.	.	.	.	.	.	E	.	.	.	.	.	.	.	.	.	.
Streptomyces seoulensis	93.9	.	.	.	.	.	.	.	.	.	.	.	.	.	.	.	.	.	.	.	.	.	.	.	.	.	.	.	.	.	.	A	.	.	.	.	.	.	.	.	.	.	.	.	.	.	.	.	.	.	.
Bacillus licheniformis	40	.	E	I	.	S	A	.	.	.	.	N	.	.	S	.	T	.	.	I	A	.	.	.	.	.	T	.	D	.	.	Y	I	S	.	E	C	.	.	E	.	.	.	K	.	.	.	.	.	S	G

Strains	PS (%)#	401*									410*										420*	
Streptomyces TML10	100	E	L	G	R	G	R	G	G	P	R	C	M	S	C	P	V	E	R	D	A	V
Streptomyces echinatus	97.3	.	.	.	.	.	.	.	.	.	.	.	.	.	.	.	.	A	.	.	.	.
Streptomyces sp. PBH53	96.1	.	.	.	.	.	.	.	.	.	.	.	.	.	.	.	.	.	.	.	.	.
Streptomyces sp. NRRL B-3648	96.1	.	.	.	.	.	.	.	.	.	.	.	.	.	.	.	.	.	.	.	P	.
Streptomyces hygroscopicus subsp. jinggangensis (strain 5008)	96.1	.	.	.	.	.	.	.	.	.	.	.	.	.	.	.	.	.	.	.	P	.
Streptomyces griseochromogenes	95.1	.	.	.	.	.	.	.	.	.	.	.	.	.	.	.	.	.	.	E	.	.
Streptomyces sp. SID4985	94.8	.	.	.	.	.	.	.	.	.	.	.	.	.	.	.	.	.	.	.	.	.
Streptomyces sp. CB01883	95.1	.	.	.	.	.	.	.	.	.	.	.	.	.	.	.	.	.	.	E	.	.
Streptomyces dangxiongensis	94.8	.	.	.	.	.	.	.	.	.	.	.	.	.	.	.	.	.	.	.	.	.
Streptomyces actinomycinicus	94.8	.	.	.	.	.	.	.	.	.	.	.	.	.	.	.	.	.	.	.	.	.
Streptomyces cellostaticus	94.6	.	.	.	.	.	.	.	.	.	.	.	.	.	.	.	.	.	.	E	.	.
Streptomyces lucensis JCM	94.6	.	.	.	.	.	.	.	.	.	.	.	.	.	.	.	.	.	.	E	.	.
Streptomyces seoulensis	93.9	.	.	.	.	.	.	.	.	.	.	.	.	.	.	.	.	.	.	.	P	.
Bacillus licheniformis	40	.	.	S	.	.	.	.	.	.	.	.	.	.	M	.	L	Y	.	E	D	.
